# Supplementary material for: Reduced injection risk behavior with co-located hepatitis C treatment at a syringe service program: The accessible care model
Source: PLoS One. 2024 Aug 29;19(8):e0308102. doi: 10.1371/journal.pone.0308102 (PMC11361571; doi:10.1371/journal.pone.0308102)
Supplement: S1 File — (PDF) [file pone.0308102.s003.pdf]

WCMC
Protocol Summary

Protocol Number: 1612017838

Status: Active - Open to Enrollment

Expiration Date: 03/19/2018

Last Approval Date:

Investigator: Marks, Kristen

Protocol Description

Review Type: Initial Protocol (Full Board or Expedited or Exempt)

Application Date: 09/29/2017

Title: Accessible Care Intervention for Engaging People who Inject Illicit Drugs (PWID) in Hepatitis C Care

Required Summary: The proposed study will examine the feasibility, acceptability, safety, effectiveness, and cost of an Accessible Care intervention for engaging people who inject illicit drugs (PWID) in hepatitis C care. Accessible Care for PWID is low-threshold care provided in programs designed specifically for PWID where they can comfortably access care without fear of shame or stigma. Accessible Care will be provided by co-locating a hepatitis treatment provider, together with a Hepatitis C Care Coordinator (HCCC), on-site at our collaborating needle exchange program. The proposed study will compare the effectiveness of Accessible Care with Usual Care (referrals to existing services) in facilitating linkage, engagement, and retention of PWID in care for hepatitis C, addiction, and HIV prevention. Our primary outcome is sustained virologic response, which constitutes virologic cure. Substance use and HIV and HCV risk behaviors are secondary outcomes. The Specific Aims of this study are to compare active PWID receiving Accessible Care or Usual Care on the following outcomes: 1. Hepatitis C Outcomes: (1a) Linkage and engagement in hepatitis C care, (1b) Successful hepatitis C treatment, (1c) Safety of hepatitis C treatment, and (1d) Rates of reinfection. 2. Substance use outcomes 3. Reductions in self-reported HIV and HCV risk behaviors. As all-oral regimens become available to patients who do not inject drugs, the substantial health disparities in hepatitis C already evident will become worse, as the haves are cured and the have-nots are not. Data are needed on effective methods to provide successful antiviral therapy to the core population affected by the epidemic, persons who are currently using illicit drugs, so that they, too, may benefit. Treatment can also help end the HCV epidemic by preventing onward transmission, but only if we have effective models for delivering treatment to people actively injecting drugs.

Performance Sites

Type	Site	Address
Performing Organization	Weill Medical College of Cornell University	Grants & Contracts 1300 York Avenue, Box 89 New York NY - 10021 USA
Performing Organization	Lower East Side Harm Reduction Center	Lower East Side Harm Reduction Center 25 Allen Street New York NY - 10002 USA

Investigators

Person Name	Primary Title	Directory Title	Units	Affiliate	Training Flag
Marks, Kristen	Assistant Professor	M.D.	Infectious Diseases	PI - Responsible for Entire Protocol	N
Kapadia, Shashi	Instructor	M.D.	Infectious Diseases	Conduct informed consent process,Screen subjects,Collect demographics,Evaluate Inclusion/Exclusion Criteria and Medical History,Perform Physical Examination,Perform other study specific interventions,Assess Adverse Events,Record Concomitant Medications,Review/Sign CRFs and/or DCFs,Protocol design,Supervision of study personnel,Data Analysis	N

Administrative Contact

Type	Name	Comments
Administrator	Marks, Kristen	

Funding Source

Type	Name/Title
Sponsor (Other)	National Institute on Drug Abuse

Actions

Description	Comments	Action Date
Expedited Approval	Amendment-002:	10/20/2017
Submitted to IRB	Amendment-002: Submit to IRB	10/09/2017
Submitted to IRB	Amendment-002: Submit to IRB	10/02/2017
Submitted to IRB	Amendment-002: Submit to IRB	09/29/2017
Approved	Amendment-001: Approval Pending changed to Approve	07/13/2017
Amendment Created	Amendment-002 created	09/29/2017
Approval Pending	Amendment-001:	07/13/2017
Approval Pending	Amendment-001: adding IRB admin: mam3012	06/23/2017
Approval Pending	Amendment-001: Submit to IRB	06/22/2017
Approval Pending	Amendment-001: Submit to IRB	06/22/2017
Approval Pending	Amendment-001: Submit to IRB	06/19/2017
Amendment Created	Amendment-001 created	06/13/2017

Actions

Description	Comments	Action Date
Administrative Correction	reupload approval	06/01/2017
Expedited Approval		05/22/2017
Submitted to IRB	Submit to IRB	05/10/2017
Submitted to IRB	Submit to IRB	05/05/2017
Submitted to IRB	Submit to IRB	04/20/2017
Modifications Required		03/20/2017
Assigned to Agenda		03/20/2017
Submitted to IRB	Submit to IRB	03/09/2017
Protocol Created	Protocol Created	12/22/2016

Amendment/ Renewal

Type	Version	Status	Created Date	Summary
Amendment	002	Approved	09/29/2017	This amendment is to 1) make minor changes requested by NYU School of Medicine IRB to the consent form (related to Dr. Ben Eckhardt¿s role as investigator, the protocol amendment to be submitted to NYU IRB along with the Weill Cornell IRB paperwork is also included for reference) 2) amend the section on subject recruitment to include active and passive recruitment from other organizations, 3) submit a study flyer to be stamped for use in this study.
Amendment	001	Approved	06/23/2017	We are submitting this amendment to add the following co-investigators: Yesenia Aponte-Melendez, Laz Davis, and Zachary Barbati (medical student). Please refer to the External Co-investigators Listing for their CITI Trainings and Study Specific Report Forms. We are updating the Risk

Amendment/ Renewal

Type	Version	Status	Created Date	Summary
				Level section to indicate that the protocol does not qualify for expedited review under any of the categories listed; it is minimal risk, but requires full board review. Therefore we changed the answer to question 3 in the Risk Level section from 'Yes' to 'No'. We are suggesting an overall increase in compensation in order to bring study incentives to levels similar to comparable studies. These changes are reflected in the consent form and in question 14 of the Subject Population section. We are also changing the entry criterion. As we were creating our eligibility checklist, we found that as worded, the current criterion do not require injection drug use within the last 90 days as we had intended (as written it could be non-injection). In addition, we removed the criterion requiring intravenous drug use for one year, which we did not deem necessary for this study. Therefore we wanted to simplify the current language for inclusion criterion, from: PWID will be recruited from among harm reduction facility participants at the Lower East Side Harm Reduction Center using

Amendment/ Renewal

Type	Version	Status	Created Date	Summary
				systematic sampling methods to recruit a representative sample of syringe exchange participants. Persons will be eligible for HCV screening if they: (1) are 18 years or older, (2) injected heroin, cocaine, or other drugs for at least 1 year, and (3) used heroin, cocaine, or other illicit drugs (excluding marijuana) in the past 90 days. To the following language: PWID will be recruited from among harm reduction facility participants at the Lower East Side Harm Reduction Center using systematic sampling methods to recruit a representative sample of syringe exchange participants. Persons will be eligible for HCV screening if they: (1) are 18 years or older, (2) injected heroin, cocaine, or other drugs in the past 90 days. This change is reflected in question 11 of the Non-Technical Research Plan section.

Additional Form/s

Protocol Number: 1612017838 **Title:** Accessible Care Intervention for Engaging People who Inject Illicit Drugs (PWID) in Hepatitis C Care **Prinicipal Investigator:** Marks, Kristen

Questionnaire Name: Subject population for Prod

Question:	Answer:
1. Will you be recruiting subjects to participate in this study? If you are obtaining tissue, examining medical records, or receiving data from another institution or database (e.g., WCMC will be acting as a coordinating center receiving data from subsites), then you must answer "yes" to this question.	Yes
2. How many subjects do you intend to enroll at WCMC (Please note, subjects are considered to be enrolled in the study once they have signed the consent form. The numbers below should include those subjects who will sign the consent form but may ultimately not participate in the study due to screen failure, not meeting inclusion/exclusion criteria, etc.)	500
3. How many subjects will be males? If male/female subjects will be enrolled regardless of gender, please indicate that here.	Participants will be enrolled regardless of gender
4. How many subjects will be females? If male/female subjects will be enrolled regardless of gender, please indicate that here.	Participants will be enrolled regardless of gender
5. What is the age range for the subjects?	over 18 without upper limit
6. What is the expected duration of study for individual subjects (days/months)? If this is a chart review or tissue procurement study, please indicate this here.	52 to 144 weeks
7. Please indicate the types of subjects you will be enrolling:	Outpatients
8. What is the subject's state of physical health? Please indicate of seriously or terminall ill.	Ambulatory subjects will be enrolled in the study. No critically ill subjects will be enrolled.
9. Will you be targeting for enrollment of any of the following special groups: Minors, Pregnant Women/Fetuses, Neonates, Students and/or employees, Prisoners, Special racial or ethnic groups, Mentally/cognitively impaired (i.e. mentally ill, mentally retarded, emotionally disturbed, senile dementia, etc.).	No
10. Please select all of the recruitment methods for initially identifying potential subjects:	Recruitment flyers/advertisements<br>Other (i.e. bedside, clinic interview, etc.)
11. Please explain Other methods (i.e. bedside, clinic interview, etc.; if you will be approaching potential subjects in the clinic or at the bedside, please indicate how potential subjects will be approached, who will introduce the study to potential subjects, and who will	The Lower East Side Harm Reduction Center is a community based syringe exchange programs in New York City. Through funding from the New York

obtain informed consent; This information should be the same in the informed consent section)	City Department of Health 'Check Hep C' program these facilities have been screening syringe exchange participants for hepatitis C. Subjects who have active hepatitis C infection through screening done by the partnering facilities will be referred to the study team for potential enrollment. Study staff will work with syringe program staff to recruit participants. Syringe program staff will refer participants from their program in person to onsite study staff. Recruitment will occur through word of mouth with planned information sessions scheduled at the syringe program's weekly hepatitis C support groups. We will also seek referrals from other organizations serving people who use drugs (methadone maintenance treatment programs, homeless services, harm reduction organizations). In partnership with those organizations, we will engage in active and passive recruitment strategies. Staff at the collaborating organization will engage in active recruitment by identifying potential participants and providing a direct referral to study staff (either by phone or using a referral form). A passive recruitment strategy will be the display of informational flyers at the collaborating sites (see attached flyer). The study flyer will include a brief description of the study, some inclusion criteria and contact information. Additional passive recruitment strategies will include posting and distributing study flyers in areas where people who use drugs are known to congregate (e.g., parks, near street drug markets).
12. If there is more than one active trial being run by the PI or in the department/division (if known), please provide an algorithm/schema or information on how it will determined which study the subject(s) will be offered. If none, state not applicable.	not applicable
13. Will subjects receive any compensation before or after study?	Yes
14. Please explain how much, at what rate, and in what form (i.e. cash, taxi fare, medical care, meals, gifts, etc.).	Participants will receive cash compensation for completing the research questionnaires (and, if applicable, providing blood) that are a part of this study, of which there will be at least four. The first research visit is the longest and is anticipated to take between 60-90 minutes. The subsequent research visits should take between 60 and 75 minutes. Participants will not be compensated for seeing the care coordinator, attending medical visits, or undergoing hepatitis C treatment. Participants will be compensated for each of the research visits which include questionnaire and, if applicable, drawing blood. The compensation schedule is as follows: $60 for first research visit; $50 for the second and third research visit (approximately at 3 and 6 months of first research visit); $60 for the forth research visit (approximately at 9 months of first research visit/interview); and $70 for the fifth research

visit (approximately at 12 months of first research visit/interview). $40 for subsequent research visits, if any. $50 for qualitative interview if the participant is asked and agrees to participate. $5 for each monthly check-in visit These compensations will be received in cash at the study visit.

Additional Form/s

Protocol Number: 1612017838 **Title:** Accessible Care Intervention for Engaging People who Inject Illicit Drugs (PWID) in Hepatitis C Care **Prinicipal Investigator:** Marks, Kristen

Questionnaire Name: Risk Level for production

Question:	Answer:
1. What is the risk level of the proposed research study?	Minimal Risk
2. Does your study qualify for exempt review in any of the categories detailed in the More section (Please click More on the right for a list of the categories)?	No
3. Does your study qualify for expedited review in any of the categories detailed in the More section (Please click More on the right for a list of the categories)?	No
4. This study is minimal risk but does not qualify for initial exempt or expedited review.	Yes

Additional Form/s

Protocol Number: 1612017838 **Title:** Accessible Care Intervention for Engaging People who Inject Illicit Drugs (PWID) in Hepatitis C Care **Prinicipal Investigator:** Marks, Kristen

Questionnaire Name: Confidentiality of Data and Privacy of Subjects

Question:	Answer:
1. Will you be collecting identifiable PHI, either from the Medical Record or during the course of the study, where the data, either directly or through a code, can be linked back to an individual?	Yes
2. Are you requesting a complete HIPAA Waiver of Authorization? If you require a waiver of HIPAA authorization for some, but not all subjects, please answer no to this question and indicate in an open text field in this section which subjects you are requesting a waiver of HIPAA authorization from (e.g., you will be obtaining authorization from some subjects, but others are lost to follow-up)(Please note, the request for the HIPAA partial waiver to confirm subject eligibility is requested in the subject population section)	No
3. Are you obtaining written HIPAA authorization from subjects by incorporating the appropriate HIPAA language into the informed consent form?	Yes
4. What specific safeguards will be employed to protect confidentiality of data?	Data is recorded both electronically and in paper format<br>Data will be coded<br>Data will be kept in the Pis locked office<br>Data will be kept on a password-protected computer<br>Data will be saved on a secure server
5. Additional Information (if any) on specific safeguards employed to protect confidentiality of data when data is recorded both electronically and in paper format.	All computerized research data will be coded, encrypted and password protected. All study participants will be assigned a unique alpha-numeric identifier at enrollment and all questionnaire data will be collected using this code. The key connecting names and study identification codes will be kept separately from any coded study data, in a locked file cabinet at the study office, and be available to study staff only. Study data in the research office will be kept in a locked cabinet or on a secure computer and will only be available to the immediate research team. No data that identify individual subjects will be made public in any reports of the results of the study. Study data will not be disclosed without the subject's permission, except in those instances where the law requires the reporting of communicable disease such as HIV or hepatitis C or in cases where the research staff has reasonable cause to suspect the occurrence of child abuse or neglect or a participant's intention to inflict violence on self or others. In these instances

	research staff will report such concerns to the appropriate resources.
6. How will samples be coded, who will have access to the code, and where will the code be kept?	All study participants will be assigned a unique alpha-numeric identifier at enrollment and all questionnaire data will be collected using this code. The key connecting names and study identification codes will be kept separately from any coded study data, in a locked file cabinet at the study office, and be available to study staff only.
7. Additional Information (if any) on specific safeguards employed to protect confidentiality of data when data is kept in the Pis locked office.	see above
8. Additional Information (if any) on specific safeguards employed to protect confidentiality of data when data is kept on a password-protected computer.	see above
9. Additional Information (if any) on specific safeguards employed to protect confidentiality of data when data is saved on a secure server.	Research data will be captured on Weill Cornell RedCaps database (for laboratory data) secure server through the Cornell Clinical Trials Unit. Questionnaire data will be captured on NDRI's secure database server.
10. Will data that identifies individual subjects be published or in any way disclosed to third parties other than project personnel or the study sponsor?	No
11. Will subjects have access to their research records while they are enrolled in the study? PLEASE NOTE: the HIPAA authorization form must include this information.	Yes

Additional Form/s

Protocol Number: 1612017838 **Title:** Accessible Care Intervention for Engaging People who Inject Illicit Drugs (PWID) in Hepatitis C Care **Prinicipal Investigator:** Marks, Kristen

Questionnaire Name: Human Tissue for Prod

Question:	Answer:
1. Tissue Submission Policy: Human tissue removed during a diagnostic or therapeutic procedure must be submitted to Pathology intact and may not be incised, opened, or damaged in any way, with the exception of surgical waste (defined below). Peripheral blood is not considered tissue. Surgical waste is specifically defined by the Medical Board as: 1. Subcutaneous tissue removed to facilitate wound closure and/or 2. Tissues significantly altered or diluted by the procedure such as lens phakoemulsifications, vitrectomy specimens or liposuction specimens. Other than the surgical waste noted above, ALL tissue must go FIRST to Pathology unless an exception to the tissue submission policy is requested. 1. Is human tissue from patients at this institution (WCMC) being used in this study based on the WCMC tissue policy described above? If yes, please complete the human tissue request form, found on our website at http://weill.cornell.edu/research/forms_and_policies/irb_forms/index.html and submit to submit2pathology@med.cornell.edu. Please note, if you answer yes to this question, IRB approval will not be released until the IRB office receives confirmation of approval from Pathology. Please check "no" if this is a review of pathology reports/results only OR tissue obtained outside WCMC-NYP.	No

Additional Form/s

Protocol Number: 1612017838 **Title:** Accessible Care Intervention for Engaging People who Inject Illicit Drugs (PWID) in Hepatitis C Care **Prinicipal Investigator:** Marks, Kristen

Questionnaire Name: Data and Safety Monitoring Plan for Prod

Question:	Answer:
1. Does this study qualify for exempt or initial expedited review?	No
2. How many study procedures are involved in this study? Note: You have to list separately all the study procedures and identify the associated risk (Minimal risk or greater than minimal risk). Study procedures would be those interventions that are occurring only because the subject is taking part in the study. This should include the administration of a study drug or an FDA-approved drug being given off label or for research purposes only. Do not include procedures that are being done as part of standard of care.	8
3. Study Related Procedure #1	After signing the consent form, participants will be asked to provide contact information, including personal telephone number and whether there are friends or family members that we can contact if we have lost contact with the participant for any reason. No personal health information will be shared with these family or friends. These persons will simply be asked to convey the message that the study team is interested in seeing the participant. Every month the participant will be asked to briefly check in with the study staff to update changes in contact information.
4. Risk Level of Procedure #1	Minimal Risk
5. Study Related Procedure #2	Questionnaires: A member of the study team will administer a detailed questionnaire at the time of enrollment into the study. This questionnaire will ask questions about demographics (e.g., age, gender, race), alcohol and drug use history and practices, sexual behavior, and experience with health care. Questionnaires will then be administered every three months for up to a 12 months , independent of whether the participant receives treatment for his or her hepatitis C, with questions about his or her substance use history and practices, sexual practices, and experience with health care
6. Risk Level of Procedure #2	Minimal Risk
7. Study Related Procedure #3	Human Immunodeficiency Virus (HIV) Testing. Participants will have a 10- to 15-minute HIV prevention counseling

session with a trained staff member. The session will cover the meaning of results from the HIV test. They will also cover how to reduce the participants' chances of being infected with HIV and other infectious diseases. The HIV test will be done by a rapid test, by sticking the tip of one finger to obtain a few drops of blood or taking an oral swab.. Participants will get counseling about what the test result means. The results will be available within about 20 minutes. Post-test counseling and referrals to service will be provided by a trained staff member. For patients living with HIV, results of a positive HIV test from the medical records may be provided instead of doing this test. Human Immunodeficiency Virus (HIV) Testing as described above will be offered at a research visit every 6 months to those participants who previously tested negative for HIV.

8. Risk Level of Procedure #3

Minimal Risk

9. Study Related Procedure #4

Data collection after randomization to Accessible Care or Usual Care arm. Participants in the Accessible Care Arm will be referred to see a study provided clinician and an Accessible Care Coordinator on site of the Lower East Side Harm Reduction Center. The hepatitis C treatment, and the medications are not being tested as part of the study. However, the response to treatment, and the laboratory tests taken as routine standard of care during treatment will be used for study purposes. Participants randomized to the Usual Care Arm will not receive on site treatment. They will be referred to a hepatitis C case manager at the harm reduction facility who will assist with referral for hepatitis C care within the community. If they start treatment for hepatitis C in the community, medical records will be requested with their permission to record the response to treatment.

10. Risk Level of Procedure #4

Minimal Risk

11. Study Related Procedure #5

(Accessible care arm only) If participants initiate treatment for hepatitis C additional 10mL of blood will be drawn at weeks 2, 4 and 12 of treatment, and 4 and 12 weeks after the end of treatment, and sent for storage. These samples may be tested looking for signs of multiple concurrent hepatitis C infections, and helping to decipher treatment failure from re-infection in participants who do not achieve cure of their hepatitis C.

12. Risk Level of Procedure #5

Minimal Risk

13. Study Related Procedure #6

(Accessible care arm only) Within 6 months of enrolling in the study, participants will

have a one-on-one meeting with a Hepatitis C coordinator to review harm reduction practices. This discussion will include discussions on prevention of hepatitis C transmission and re-infection, ways to prevent illicit drug overdose, options for medication-assisted treatment for opioid addiction, among others.

14. Risk Level of Procedure #6

Minimal Risk

15. Study Related Procedure #7

Assessment for reinfection: Participants that achieve a sustained virologic response after completing hepatitis C treatment will be given an appointment to come back every three months to complete a study interview and be tested for hepatitis C, for up to 144 weeks after treatment completion.

16. Risk Level of Procedure #7

Minimal Risk

17. Study Related Procedure #8

Qualitative interviews: During the study, some participants will be asked to take part in a 60-90-minute, digitally audio-recorded interview. If they choose to participate in the interview, they will be asked open ended questions about: health care and hepatitis C-related healthcare experiences, alcohol and drug use, injection risk behaviors and sexual practices. They will be asked how these changed, if they did, during study participation.

18. Risk Level of Procedure #8

Minimal Risk

19. What data will be collected during the course of the study to assess both safety and efficacy?

DSMB reports will include number of participants that enrolled, were treated for HCV, that discontinued study and that were lost to follow up (by study arm). Our expected rate of enrollment is 100 subjects per year. Efficacy Endpoint: Sustained Viriologic Responses = HCV cure (the primary end point) Safety endpoints: 1. Hospitalizations or Deaths related to Overdose 2. Overall Deaths 3. Severe adverse events (Defined as Severe or medically significant, but not immediately life-threatening; hospitalization or prolongation of hospitalization indicated; disabling; limiting self care activities of daily living. (Grade 3); Life-threatening consequences; urgent intervention indicated (Grade 4); Death related AE (Grade 5)).

20. Describe the interim and complete stopping rules for this study. If there are no defined stopping rules, please provide justification for why there are none.

Efficacy Endpoints: We anticipate significantly more HCV treatment and thus more Sustained Viriologic Responses (SVRs, the primary end point) in Accessible Care compared to Usual Care arm; however, the results of several important secondary outcomes (e.g. cost, reinfections, changes in drug use, etc) are also required to determine if this strategy is implementable. Therefore, we have not included an interim analysis

to compare the SVR rate between the intervention and control arms. If contrary to what we expect, there is no difference in treatment uptake between the Accessible Care and Usual Care arms, the data will be analyzed for the secondary endpoints (reinfection, safety, progression through care cascade, etc). Safety endpoints: 1. Hospitalizations or Deaths related to Overdose 2. Overall Deaths Will be compared annually (after first 30 treated in Accessible Care arm) between Accessible care and Usual Care arms. An increase of 20% in Hospitalizations or Deaths related to Overdose in the intervention arm would necessitate a halt in enrollment and review of the study. An increase of 20% in Overall Deaths in the intervention arm would necessitate a halt in enrollment and review of the study. 3. SAEs (Defined as Severe or medically significant, but not immediately life-threatening; hospitalization or prolongation of hospitalization indicated; disabling; limiting self care activities of daily living. (Grade 3); Life-threatening consequences; urgent intervention indicated (Grade 4); Death related AE (Grade 5)). Given limited data on the use of these regimens in active drug users, all SAEs that occurring during HCV treatment period (during treatment plus 30 days after) in the intervention arm will be included in the DSMB report. Attribution (related or not-related) to HCV treatment will be made by the investigators. In addition, all SAEs (collected at the 3 month interviews) will be reported for each arm as a point of reference for the general frequency of SAEs in this population. Because we anticipate low treatment rates in the control arm, the stopping rules for HCV Treatment-related SAEs will be based on the Accessible Care arm alone. If 20% of participants treated in the Accessible Care arm (after first 30 treated) experience Treatment related-SAEs, enrollment will be halted and a review of the study initiated. All unexpected HCV treatment-related SAEs will be reported to the IRB and evaluated for impact on study (consent etc.) as per standard in real time.

21. Describe what adverse events may cause termination/dropout of a subject.	As above, to repeat: Safety endpoints: 1. Hospitalizations or Deaths related to Overdose 2. Overall Deaths 3. SAEs (Defined as Severe or medically significant, but not immediately life-threatening; hospitalization or prolongation of hospitalization indicated; disabling; limiting self care activities of daily living. (Grade 3); Life-threatening consequences; urgent intervention indicated (Grade 4); Death related AE (Grade 5)).
22. Describe the anticipated adverse events and risks of study intervention(s), agent(s) and/or device(s) in detail (e.g., potential risk of the study drug, including potential drug interactions, psychological	Rationale for Efficacy and Safety Endpoints: The intervention in this study is the site of HCV care (not the treatment itself). We anticipate that providing HCV care

risks; physical risks). Assess their seriousness and incidence of complications or adverse events when known. Include animal data if trials in humans have not been performed.

and treatment on site at a community syringe exchange center will allow access to treatment for greater numbers of people who inject drugs (PWID). Treatment on this study will include FDA-approved standard of care regimens with excellent safety profile; however limited data exists on the use of these agents in PWID. While experts have little concern for drug-drug interactions between most DAAs and illicit drugs, we have chosen to monitor HCV Treatment-related SAEs, Hospitalizations and Deaths related to Overdose, and Overall Deaths as safety endpoints that would trigger review of the study. Other on treatment side effects will also be captured and described, but will not impact conduct of the study and thus will not be reported to DSMB. Currently, HCV cure rates exceed 90% with currently available all oral DAA regimens in clinical trials and recent clinical cohorts regardless of HCV genotype; however little is known about treatment efficacy and safety in active drug users as these patients are typically excluded from clinical trials. One clinical trial of a modern era DAA regimen was conducted in 301 persons receiving opioid agonist therapy (OAT) (Merck C-EDGE Co-STAR) and provides some safety and efficacy data relevant to our study. It did not exclude patients who were actively using drugs of potential abuse, and more than 50% of patients had at least one urine drug screen positive for potential drugs of abuse. The study randomized patients to the fixed dose combination of elbasvir/grazoprevir or placebo, so that safety could be carefully ascertained in this patient population perceived as being high risk for unrelated adverse events. Those that received placebo were subsequently treated open label. The primary efficacy outcome showed an SVR 12 of 91.5% in the immediate treatment group and 89.5% in the delayed treatment group. Positive urine drug screens did not affect adherence or efficacy. 18 patients had recurrent viremia and 5 were due to probable reinfection. The safety profile was similar between treatment and placebo with rare SAEs (3.5% in treated, 4% in placebo); however, only 1 SAE in each group was considered treatment-related. The high efficacy and relative safety of this study of patients on OAT, many of who also used drugs of potential abuse, is reassuring that HCV treatment may be safely delivered to our study population.

23. Given the risks identified, describe what monitoring is needed to immediately recognize the adverse events that do occur.

Monitoring for previously mentioned severe adverse events will be accessed at scheduled medical visits at week 2, 4, 8, and 12 of treatment. Additionally participants will be encouraged to contact medical or research staff with any potential adverse events in the intervening times.

24. Describe what measures will be taken to minimize any negative impact on subjects resulting from study closure or a subject being terminated from the study.

Recruitment of new participants will end several months before study closure to try and limit any potential negative impacts from study closure. For participants who are being worked up for potential HCV treatment, or currently receiving treatment or immediate follow-up will have arrangements for their care to be completed with partnering Infectious Diseases provider (either through Weill Cornell Medicine or NYU School of Medicine).

25. Describe the adverse event grading based on severity, attribution, and expectedness (including frequency) that will be used, to whom they will be reported and how often.

See above, to repeat: Safety endpoints: 1. Hospitalizations or Deaths related to Overdose 2. Overall Deaths Will be compared annually (after first 30 treated in Accessible Care arm) between Accessible care and Usual Care arms. An increase of 20% in Hospitalizations or Deaths related to Overdose in the intervention arm would necessitate a halt in enrollment and review of the study. An increase of 20% in Overall Deaths in the intervention arm would necessitate a halt in enrollment and review of the study. 3. SAEs (Defined as Severe or medically significant, but not immediately life-threatening; hospitalization or prolongation of hospitalization indicated; disabling; limiting self care activities of daily living. (Grade 3); Life-threatening consequences; urgent intervention indicated (Grade 4); Death related AE (Grade 5)). In addition, Given limited data on the use of these regimens in active drug users, all SAEs that occurring during HCV treatment period (during treatment plus 30 days after) in the intervention arm will be included in the DSMB report. Attribution (related or not-related) to HCV treatment will be made by the investigators. In addition, all SAEs (collected at the 3 month interviews) will be reported for each arm as a point of reference for the general frequency of SAEs in this population. Because we anticipate low treatment rates in the control arm, the stopping rules for HCV Treatment-related SAEs will be based on the Accessible Care arm alone. If 20% of participants treated in the Accessible Care arm (after first 30 treated) experience Treatment related-SAEs, enrollment will be halted and a review of the study initiated. All unexpected HCV treatment-related SAEs will be reported to the IRB and evaluated for impact on study (consent etc.) as per standard in real time.

26. In addition to the IRB, specify to whom you will be reporting unexpected adverse events to promptly. PLEASE NOTE all reportable AEs should be reported to the WCMC IRB as per WCMC AE reporting policy.

A data safety monitoring board will review this plan, interim data, and additionally review any unexpected adverse events. There is no sponspor for whom immediate reporting is required.

27. Will you be using a medical monitor?

Yes

28. Please select the appropriate affiliation of the monitor:

	Affiliated with WCMC-NYP and is a part of the research study.
29. Will interim analysis be performed?	Yes
30. Please specify at what intervals the interim analysis will be performed.	Interim safety endpoints will be compared after first 30 treated in Accessible Care arm and then annually, between Accessible care and Usual Care arms.
31. Please specify to whom the data will be reported.	Data will be reported to the Data Safety Monitoring Board
32. Will you be utilizing a Data Safety Monitoring Board (DSMB) or Data Monitoring Committee (DMC)?	Yes
33. Will you be using the WCMC DSMB?	Yes
34. Please specify what will be reported to the DSMB/DMC.	DSMB reports will include number of participants that enrolled, were treated for HCV, that discontinued study and that were lost to follow up (by study arm). Our expected rate of enrollment is 100 subjects per year. Efficacy Endpoints We anticipate significantly more HCV treatment and thus more Sustained Viriologic Responses (SVRs, the primary end point) in Accessible Care compared to Usual Care arm; however, the results of several important secondary outcomes (e.g. cost, reinfections, changes in drug use, etc) are also required to determine if this strategy is implementable. Therefore, we have not included an interim analysis to compare the SVR rate between the intervention and control arms. If contrary to what we expect, there is no difference in treatment uptake between the Accessible Care and Usual Care arms, the data will be analyzed for the secondary endpoints (reinfection, safety, progression through care cascade, etc). Safety endpoints: 1. Hospitalizations or Deaths related to Overdose 2. Overall Deaths Will be compared annually (after first 30 treated in Accessible Care arm) between Accessible care and Usual Care arms. An increase of 20% in Hospitalizations or Deaths related to Overdose in the intervention arm would necessitate a halt in enrollment and review of the study. An increase of 20% in Overall Deaths in the intervention arm would necessitate a halt in enrollment and review of the study. 3. SAEs (Defined as Severe or medically significant, but not immediately life-threatening; hospitalization or prolongation of hospitalization indicated; disabling; limiting self care activities of daily living. (Grade 3); Life-threatening consequences; urgent intervention indicated (Grade 4); Death related AE (Grade 5)). In addition, Given limited data on the use of these regimens in active drug users, all SAEs that occurring during HCV treatment period (during treatment plus 30 days after)

	in the intervention arm will be included in the DSMB report. Attribution (related or not-related) to HCV treatment will be made by the investigators. In addition, all SAEs (collected at the 3 month interviews) will be reported for each arm as a point of reference for the general frequency of SAEs in this population. Because we anticipate low treatment rates in the control arm, the stopping rules for HCV Treatment-related SAEs will be based on the Accessible Care arm alone. If 20% of participants treated in the Accessible Care arm (after first 30 treated) experience Treatment related-SAEs, enrollment will be halted and a review of the study initiated. All unexpected HCV treatment-related SAEs will be reported to the IRB and evaluated for impact on study (consent etc.) as per standard in real time.
35. Please specify how often these reports will be made to the DSMB/DMC.	Reports will be presented annually (after first 30 treated in Accessible Care arm) between Accessible care and Usual Care arms.

Additional Form/s

**Protocol Number:**	1612017838	**Title:**	Accessible Care Intervention for Engaging People who Inject Illicit Drugs (PWID) in Hepatitis C Care	**Prinicipal Investigator:**	Marks, Kristen

Questionnaire Name: Use of Drugs or Biological Agents

Question:	Answer:
1. Does this study involve the administration of an FDA regulated product, Nutritional supplement or a biological product?	No

Additional Form/s

Protocol Number: 1612017838 **Title:** Accessible Care Intervention for Engaging People who Inject Illicit Drugs (PWID) in Hepatitis C Care **Prinicipal Investigator:** Marks, Kristen

Questionnaire Name: Non-Technical Research Plan Prod

Question:	Answer:
1. What is the expected end date of the study? Any information that is written in this space prior to CSEC approval will be overwritten by the CSEC upon approval.	10/01/2021
2. Will there be student investigators (must be older than 18 years of age)? Any information that is written in this space prior to CSEC approval will be overwritten by the CSEC upon approval.	Yes
3. List the student names. Any information that is written in this space prior to CSEC approval will be overwritten by the CSEC upon approval.	Zachary Barbati
4. What are their responsibilities in the project? Any information that is written in this space prior to CSEC approval will be overwritten by the CSEC upon approval.	Zachary will be a Volunteer Research Assistant in this study. His main role will be to act as link between the existing services within the community based organization and our research study. Specifically, the Lower East Side Harm Reduction Center (through State and City funding) has an extensive HCV screening program. Zachary will help in the linkage of HCV antibody positive participants potentially interested in our study link with a member of our study team for consideration of enrollment. Conversely when one of our study participants expresses a need that can be met through existing study services (such as housing assistance, substance use treatment, insurance enrollment, etc.), Zachary will help ensure linkage from our research team to the appropriate Harm Reduction Center representative. Additionally, Zachary will 1) assist in contacting study participants, both as a check-in and as reminders for upcoming research visit, 2) aid in data entry, 3) interface with laboratory and pharmacy representative, and 4) help escort participants to appointments outside the center (for instance for Liver Ultrasound testing) when needed.
5. Please list the investigator(s) who will be supervising the students. Any information that is written in this space prior to CSEC approval will be overwritten by the CSEC upon approval.	Marks, Kristen
6. Study Design: Include information on the hypothesis, research question, standard vs. experimental procedures (interventions	The proposed study will compare the effectiveness of Accessible Care with Usual Care in facilitating linkage, engagement,

happening as part of clinical care vs. those that are occurring only because the subject is part of the study), the use special or unusual equipment or procedures.Include specifics on all study interventions and their frequency and the treatment plan (For example, the dosage of a drug to be given and the frequency). For randomized studies, list the study groups and under each describe the categories of procedures. List together in a group all procedures that are part of standard of care treatment,and list together in a group all procedures that are investigative, separating and labeling the two groups. Tables and/or charts are helpful and encouraged and should be uploaded in the attachments section as a continuation of the study design. Any information that is written in this space prior to CSEC approval will be overwritten by the CSEC upon approval.

and retention of people who inject drugs (PWID) in care for hepatitis C, addiction, and HIV prevention. "Accessible Care" for PWID includes features such as an informal, nonjudgmental atmosphere, availability of walk-in appointments, and a harm reduction framework to help them identify and pursue their own personal health goals. Accessible Care will be provided by co-locating a hepatitis treatment provider, together with a Hepatitis C Care Coordinator, on-site at our collaborating needle exchange program. "Usual care" represents the current procedure after someone tests positive for HCV antibody on site at the syringe exchange program. An on site care coordinator (not provided by study) provides assists with insurance and linkage to HCV providers through the NYC Dept of Health Check Hep C program. We will enroll 300 HCV(+) PWID at a needle exchange program in the Lower East Side, Manhattan and randomize them 1:1 to receive Accessible Care or Usual Care. Participants randomized to Accessible Care will receive on-site HCV care and treatment and the needle exchange program by a study-provided clinician and care coordinator. Participants in the Usual Care arm will receive referrals to existing services only. Using intent to treat methods, we will compare the proportion of participants in each arm who achieve linkage, engagement, and retention in care for hepatitis C, addiction, and HIV prevention during 12 months of follow-up. Our primary outcome is sustained virologic response (SVR), which constitutes virologic cure. Changes in substance use and HIV and HCV risk behaviors are secondary outcomes. The Specific Aims of this study are to compare active PWID receiving Accessible Care or Usual Care on the following outcomes: 1. Hepatitis C Outcomes 1a. Linkage and engagement in hepatitis C care, assessed as (a) stated interest in hepatitis C treatment evaluation, (b) attendance at an initial visit with a hepatitis provider, (c) completion of a medical evaluation for antiviral treatment, and (d) attendance at two visits with a hepatitis treatment provider within 3 months. 1b. Successful hepatitis C treatment, defined as (e) beginning antiviral treatment, (f) adherence to antiviral treatment, (g) completion of antiviral treatment, and (h) sustained virologic response, the primary outcome. We will also describe (i) the reasons for any participants not reaching treatment endpoints (a)-(h) according to staff and participant report, and (i) the costs of the intervention in each arm. 1c. Safety of hepatitis C treatment measured by the frequency of side effects, adverse events, and dose- limiting toxicity, since these rates haven't been reported in PWID receiving all-oral regimens. 1d. Rates of reinfection in active PWID who have achieved SVRs with antiviral therapy for hepatitis C. Reinfection

is one of the primary reasons cited for withholding antiviral treatment from PWID. 2. Substance use outcomes 2a. Substance abuse treatment entry and retention in each arm 3, 6, and 9 months after randomization. 2b. Reductions in substance use during follow-up assessed as (a) self-reported frequency of drug injection, and (b) self-reported percentage of days of drug or alcohol use, and (c) urine toxicology. 3. Reductions in self-reported HIV and HCV risk behaviors, namely, the frequency of high-risk injection practices and sexual risk behaviors in each arm 3, 6, and 9 months after randomization. Participants in both arms will continue to receive harm reduction services at our collaborating needle exchange program where they were enrolled. Syringe exchange, together with substance abuse treatment, are two of the most effective HIV and HCV prevention services for PWID and represent the standard of care. The study intervention will build on this HIV infection and HCV reinfection prevention support.

7. Rationale and Justification for the study: for example, historical background, investigator's personal experience, pertinent medical literature. Please include any information regarding studies in animals that are pertinent to the proposed study. If this study involves an investigational drug, an FDA approved drug being used off label or that is being given according to label but for research purposes only, please indicate what are the effects of the drug for its intended use (dosage range and efficacy, data in humans plus animal studies, when appropriate. Any information that is written in this space prior to CSEC approval will be overwritten by the CSEC upon approval.

The proposed study will examine the feasibility, acceptability, safety, effectiveness, and cost of an Accessible Care intervention for engaging people who inject illicit drugs (PWID) in hepatitis C care. Four times as prevalent in the US as HIV infection, hepatitis C is already the leading cause of liver failure and liver transplantation, and the disease burden and health care costs will continue to rise in the coming decades. The 1.5-2.0 million PWID constitute the core of the hepatitis C epidemic in the United States, with prevalence rates >70% in most studies. New, all-oral antiviral treatment regimens can eradicate HCV in nearly 100% of previously untreated patients. Nonetheless, few studies have reported successful treatment of this infection in active PWID - none with the new all-oral regimens - and they are rarely offered antiviral treatment. As more advantaged populations gain access to the treatments and are cured, health disparities in hepatitis C outcomes will sharpen. Obstacles to engaging active PWID in care include their competing priorities (e.g., legal, financial, interpersonal, drug-related, housing), more pressing medical or social problems, comorbid psychiatric illness, addiction, fear of stigma, and historically poor relations with health care providers. Other barriers exist at the provider, healthcare system, and structural levels. Accessible Care for PWID is low-threshold care provided in programs designed specifically for PWID, in community-based locations where they can comfortably access care without fear of shame or stigma. In collaboration with community-based organizations providing services to PWID in the South Bronx, East Harlem, and the Lower East Side of

Manhattan, we developed an Accessible Care program and found that we could effectively treat both hepatitis C and addiction in a sample of PWID recruited from community-based settings who were actively using illicit drugs. By reducing illicit drug use and providing effective HIV and HCV prevention, the intervention reduced HIV risk behavior as well - a finding supported by a low HCV reinfection rate in follow-up. The proposed study will compare the effectiveness of Accessible Care with Usual Care in facilitating linkage, engagement, and retention of PWID in care for hepatitis C, addiction, and HIV prevention. We will enroll 300 HCV(+) PWID at a needle exchange program in the Lower East Side of Manhattan and Bushwick neighborhood in Brooklyn and randomize them 1:1 to receive Accessible Care or Usual Care. Patients in the Usual Care arm will receive referrals to existing services at the syringe exchange program only. Using intent to treat methods, we will compare the proportion of participants in each arm who achieve linkage, engagement, and retention in care for hepatitis C, addiction, and HIV prevention during 9 months of follow-up. Our primary outcome is sustained virologic response (SVR), which constitutes virologic cure. Substance use and HIV and HCV risk behaviors are secondary outcomes. The Specific Aims of this study are to compare active PWID receiving Accessible Care or Usual Care on the following outcomes:

8. Primary Objective: Any information that is written in this space prior to CSEC approval will be overwritten by the CSEC upon approval.

The proposed study will compare the effectiveness of Accessible Care with Usual Care in facilitating linkage, engagement, and retention of PWID in care for hepatitis C, addiction, and HIV prevention. Our primary outcome is to evaluate the difference in sustained virologic response (SVR), which constitutes virologic cure, between the two study arms. SVR indicates the elimination of HCV infection and the cessation of the hepatitis disease process, and is universally considered the ultimate goal of treatment. Definitions of Accessible and Usual Care: "Accessible Care" for PWID is low-threshold care provided in the needle exchange programs, where they can comfortably access services without fear of the shame or stigma that often attends them in mainstream institutions. It includes features such as an informal, nonjudgmental atmosphere, availability of walk-in appointments, and a harm reduction framework to help them identify and pursue their own personal health goals. Accessible Care will be provided by co-locating a hepatitis treatment provider, together with a Hepatitis C Care Coordinator, on-site at our collaborating needle exchange program. Usual care represents the current procedure after someone tests positive for HCV antibody on site at the syringe exchange program. An on site care coordinator (not

provided by study) assists with insurance and linkage to HCV medical provider at sites throughout NYC through the NYC Dept of Health Check Hep C program.

9. Secondary Objective: Any information that is written in this space prior to CSEC approval will be overwritten by the CSEC upon approval.

Study Objective 1: Hepatitis C Outcomes. We have chosen a series of 8 hepatitis C outcomes that represent the continuum of care - 4 assessing linkage and engagement, and 4 assessing successful treatment. Outcomes will be compared using intent-to-treat analyses, which classify participants lost to follow-up as not having reached any unmeasured endpoint. SVR, the eighth and last of these outcomes, is the primary endpoint. Seven proximal hepatitis C endpoints have been chosen, however, so that if the intervention is not ultimately successful in achieving SVRs we can examine at what stage the failures occurred. 1a. Hepatitis C treatment engagement. We will assess the proportion of patients in each arm who (a) affirm a desire for hepatitis C treatment, (b) attend an initial visit with a hepatitis treatment provider, (c) complete a medical evaluation for antiviral treatment, including a history, physical examination, and laboratory evaluation, and (d) attend two visits with a hepatitis C treatment provider within 3 months of randomization. Hepatitis C treatment success. The four endpoints will be defined as follows. (a) Antiviral treatment initiation will be defined as taking the first dose of antiviral medication within 3 months of randomization. (b) Adherence to antiviral treatment will be assessed by self-report and calculated as a percentage of prescribed doses. (c) Treatment completion will be defined as taking the prescribed medications for the entire prescribed treatment duration. (d) Sustained virologic response (SVR) will be defined as testing HCV RNA negative 12 weeks after treatment completion, the standard definition. 1b. Cost of intervention: The HCCC and the Clinician will log time spent providing services, excluding research activities. Costs will be assigned to staff time based on prevailing national wage rates. Patient time costs will also be estimated based on time spent with staff members and will be valued based on the minimum wage because most patients are likely to be unemployed. We will describe patient-reported health care visits, employment, and incarceration that will be used as inputs to future cost or cost-effectiveness studies. 1c. Safety: Because concern about side effects has been cited as a reason not to treat PWID for hepatitis C, and because experience with these drugs in PWID has not been reported, we will report prospectively collected data on safety outcomes on all participants who receive antiviral therapy. All treatment-emergent side effects (e.g., fatigue, headache, nausea, insomnia, irritability, anorexia, diarrhea, constipation, etc.) will be

tabulated, as will adverse events, including abnormalities in serum bilirubin, liver enzymes, infections, hospitalizations, and deaths), and any adverse effects requiring treatment discontinuation, according to OHRP and NIH guidelines. We will describe the frequency of side effects, adverse events, and treatment-limiting outcomes, among all study participants who received at least one dose of antiviral medication. If numbers permit, we will compare the rates in the two arms to see whether AC influences perceived side effects. 1d. Reinfection: We will report reinfection rates in all patients in both arms who achieve SVR using time-to- event methods. ========== Study Objective 2: Substance use. We will report rates of addiction treatment entry and retention; changes in levels of self-reported alcohol and illicit drug consumption. Outcomes will be examined at 3, 6, and 9 months after randomization, to assess the magnitude and durability of intervention effects.

10. Statistical Considerations (e.g. justification for sample size or "n". Please include the total number of subjects to be recruited at WCMC, the total number of subjects at all site (if a multisite study), expected total screening failures/dropouts at WCMC (if applicable), How the data will be analyzed, etc: Any information that is written in this space prior to CSEC approval will be overwritten by the CSEC upon approval.

Using standard methods for power calculations and alpha=.05, a randomized trial comparing two arms with 150 participants in each arm will have 80% power to detect the differences in proportions of study endpoints in the two arms. This power will be sufficient to detect meaningful differences between the study arms and achieve our study aims. For example, we will have 80% power to detect a difference if 66% of participants initiate antiviral therapy in an intervention arm and 50% in the control arm do. Differences between the arms of 20% or more in any outcome will be detectable. These are clinically meaningful differences and are conservative estimates of our anticipated effect sizes. All patient will be recruited from Lower East Side Harm Reduction Center. Combined these two facilities engage about 3000 injection drug users annually with a citywide seroprevalence of HCV in PWID of 40% based on the 2015 Injection Drug Users Health Alliance Survey (about 1200 HCV(+) PWID accessing one of the two sites).

11. Inclusion Criteria: Any information that is written in this space prior to CSEC approval will be overwritten by the CSEC upon approval.

PWID will be recruited from among harm reduction facility participants at the Lower East Side Harm Reduction Center using systematic sampling methods to recruit a representative sample of syringe exchange participants. Persons will be eligible for HCV screening if they: (1) are 18 years or older, (2) injected heroin, cocaine, or other drugs in the past 90 days. Eligible persons will be tested for HCV using rapid testing methods (OraQuick HCV Rapid Antibody Test, Bethlehem, PA). Staff experienced in drawing blood from long-term PWID who have poor venous access will draw venous blood from persons testing HCV antibody positive for HCV RNA confirmatory testing. Persons who meet the

screening eligibility criteria listed above, test HCV RNA positive, and provide written consent (including consent for researchers to examine their hepatitis C medical records) will be eligible to enroll in the randomized clinical trial. Accounting for persons who test HCV-negative, are in care, or decline to participate, we estimate that approximately 500 persons will be screened during Years 01-03 to accrue a total of 300 eligible, consenting clinical trial participants.

12. Exclusion Criteria: Any information that is written in this space prior to CSEC approval will be overwritten by the CSEC upon approval.	Persons already in care for hepatitis C, defined as having had at least 2 visits with a hepatitis treatment provider within the past 6 months, will be excluded. Patients with decompensated cirrhosis will be excluded.
13. Will you be collecting as a part of this research study any of the following: tissue from surgical pathology, blood, urine, bone marrow aspirate or other biological samples?	Yes
14. Select the type of Bodily fluid collected	Blood
15. Blood Amount	5mL
16. Blood Frequency	Blood for research purposes will be drawn from each participant at their initial research visit. Participants in the accessible care arm of the study will have additional research blood drawn (at the same time as the standard of care labs are drawn) while participants are receiving hepatitis C treatment at week 2, 4, 12 of treatment. After the completion of treatment participants will have research blood drawn at 4 and 12 weeks after completion of therapy.
17. Will any genetic analysis be performed on the collected tissue?	No
18. Will any data, tissue, blood and/or other biological sample(s) be collected/stored for use in future research? The HIPAA authorization form should contain the same information as in this section.	Yes
19. Where will the data, tissue, blood and/or other biological sample(s) be stored/ sent? Please check all that apply.	WCMC Research Repository/Database
20. Could any unspecified genetic tests be done on these stored samples? If you select Yes then please include the applicable sections of the genetic testing consent form in your consent form.	No
21. Does the research planned in this project involve any form of invasive procedure (minimally invasive or greater, including venipuncture)?	Yes
22. Will any of these procedures be done for research purposes only? Answering No to this question implies all procedures are being done as standard of care.	Yes

23. Will the procedure take place in the NYP surgical suite/operating room?	No
24. Where will the procedure take place?	Research blood draws will take place at the community based research site (Lower East Side Harm Reduction Center).
25. Will you require the services of anesthesiology?	No
26. Does the research planned in this project involve any major changes in diet or exercise?	No
27. Does the research planned in this project involve any administration of physical stimuli other than auditory and visual stimuli associated with normal classroom situations?	No
28. Does the research planned in this project involve any deprivation of physiological requirements such as nutrition or sleep, manipulation of psychological and/or social variables (i.e. sensory deprivation, social isolation, psychological stress, etc.?	No
29. Does the research planned in this project involve any use of deceptive techniques without the knowledge of the subect?	No
30. Does the research planned in this project involve any probing of information which might be considered personal or sensitive, including the examination of the medical record?	Yes
31. Please explain in detail.	Study participants may feel uncomfortable responding to questions about their drug use and illegal or high-risk behavior or learning about their medical condition. Some of the questions and discussions may make subjects feel guilty, anxious, or embarrassed. Study participants may feel worried or depressed when they find out or are reminded about their risk of hepatitis C. Those who test positive could experience fear, shock, anxiety, or depression when they learn the results of their tests. Participants are free not to answer questions or talk about anything that makes them uncomfortable. They may terminate their participation in the study at any time. Participants will be assured that the information given is confidential and that interviewers are not passing judgement on any activities they report. Study staff are available to talk with participants who become anxious, worried, sad, depressed, or uncomfortable as a result of participating in the study. Referrals for mental health services will be made as needed.
32. Does the research planned in this project involve any presentation to the subject of any materials which they might find to be be offensive, threatening or degrading?	No

Additional Form/s

**Protocol Number:**	1612017838	**Title:**	Accessible Care Intervention for Engaging People who Inject Illicit Drugs (PWID) in Hepatitis C Care	**Prinicipal Investigator:**	Marks, Kristen

Questionnaire Name: Radiation Safety for Prod

**Question:**	**Answer:**
1. Will this study involve in vivo imaging or image guided interventions (e.g., CT, US, MRI, x-ray, fluoroscopy, etc.)?	No
2. Will this study involve the use of radioisotopes or other sources of ionizing radiation (i.e., diagnostic and/or therapeutic radiation, e.g. xray machines, CT, cardiac catheterization, radioisotopes, radiation therapy, etc.) for purposes other than standard of care either entirely or in part? (i.e., subjects would not be having these procedures in the manner described in the protocol and informed consent if they were not enrolled in the study; or subjects might have some of these procedures for standard of care and some of these procedures outside of normal standard of care)? If so, please obtain review and approval from the Radiation Safety Committee and attach the approval documentation to the eIRB application prior to submission. You must receive approval from the Radiation Safety Committee (RSC) before IRB approval can be released. Contact the RSC Chair, Stanley Goldsmith, M.D. at 212-746-4588 or sjg2002@med.cornell.edu for more information. Please contact Peter Capitelli (pec2008@med.cornell.edu) the Radiation Safety Officer, for assistance in calculating the dosimetry prior to submitting the protocol to the IRB or Radiology for review.	No

Additional Form/s

**Protocol Number:**	1612017838	**Title:**	Accessible Care Intervention for Engaging People who Inject Illicit Drugs (PWID) in Hepatitis C Care	**Prinicipal Investigator:**	Marks, Kristen

Questionnaire Name: Test Articles and Bioavailability/Bioequivalence S

Question:	Answer:
1. Will this study involve the use of test articles and/or is a bioavailability/bioequivalence study?	No

Additional Form/s

Protocol Number: 1612017838 **Title:** Accessible Care Intervention for Engaging People who Inject Illicit Drugs (PWID) in Hepatitis C Care **Prinicipal Investigator:** Marks, Kristen

Questionnaire Name: Additional Information for prod

Question:	Answer:
1. Will this study take place in WCMC/NYP?	No
2. Will this study take place in a private office or another location outside of WCMC/NYP?	Yes
3. Please provide the location and address of where this study will be taking place.	The study will take place at the office of Lower East Side Harm Reduction Center (25 Allen St, New York, NY 10002).
4. Please indicate the number of externally funded research and/or sponsored projects that this IRB protocol falls under. If this protocol is funded internally please enter 0. Externally funded research and/or sponsored projects include the following. Please click "More" for definitions of these terms. A) Grant/Contract/Subaward/Clinical Trial Grant B) Clinical Trial Agreement (Investigator Initiated) C) Clinical Trial Agreement (Industry Initiated) D) Sponsored Research Agreement (Investigator Initiated) E) Sponsored Research Agreement (Industry Initiated) F) Material Transfer Agreement (Recipient Initiated) G) Material Transfer Agreement (Provider Initiated) H) Other (i.e. Data Use Agreement, Registry)	1
5. Project 1: Please select the type of project this protocol falls under.	Grant/Contract/Subaward/Clinical Trial Grant
6. Start date for Grant/Contract/Subaward/Clinical Trial Grant	07/01/2016
7. End date for Grant/Contract/Subaward/Clinical Trial Grant	06/30/2021
8. What is the grant number/sponsor protocol number?	R01DA041298
9. What is the project title?	Accessible Care Intervention for Engaging People who Inject Illicit Drugs (PWID) in Hepatitis C Care
10. What is the sponsor name?	National Institute on Drug Abuse (NIDA)
11. For grants, enter the submission date. For other project types, enter N/A.	05/2015

Additional Form/s

**Protocol Number:**	1612017838	**Title:**	Accessible Care Intervention for Engaging People who Inject Illicit Drugs (PWID) in Hepatitis C Care	**Prinicipal Investigator:**	Marks, Kristen

Questionnaire Name: Potential Benefits for Prod

**Question:**	**Answer:**
1. Please assess potential benefits to subjects and to society which may accrue as a result of this research, analyzing the risk/benefit ratio and why the risks are justified by the potential benefits.	People who inject drugs are the risk group most likely to be infected with HCV, and the least likely to be offered treatment. This study will offer important insights into the feasibility, safety and efficacy of treating active drug users using a co-located model of care designed with their needs in mind. Identifying effective ways to approach HCV treatment in this marginalized population will improve overall health and quality of life and reduce the spread of this disease. Participation in the study may benefit participants by eliminating the hepatitis C virus or halting the progression of this disease. Subjects may also benefit from the discontinuation of illicit drug use through our treatment model and improved health status and quality of life. Serious adverse events related to HCV antiviral treatment are uncommon. Should they occur the close monitoring structure allows early detection and intervention and the timely provision of expert medical care. Given the low risk presented by participation in this study, the risk/benefit ratio is very favorable.

Additional Form/s

Protocol Number: 1612017838 **Title:** Accessible Care Intervention for Engaging People who Inject Illicit Drugs (PWID) in Hepatitis C Care **Prinicipal Investigator:** Marks, Kristen

Questionnaire Name: Institutional Biosafety Committee for Prod

Question:	Answer:
1. Will human research subjects be exposed to the deliberate transfer of either recombinant nucleic acid molecules, DNA or RNA derived from recombinant nucleic acid molecules, or synthetic nucleic acid molecules (Human Gene Transfer)?	No
2. Does this study involve the use of non-FDA approved biological agents in human subjects, i.e., use of a biological agent that has the ability to replicate or potentially cause disease in humans (virus, bacteria, vaccine or etiological agent)?	No

Additional Form/s

Protocol Number: 1612017838 **Title:** Accessible Care Intervention for Engaging People who Inject Illicit Drugs (PWID) in Hepatitis C Care **Prinicipal Investigator:** Marks, Kristen

Questionnaire Name: Clinical Translational Science Center for Prod

Question:	Answer:
1. Will the clinical Translational Science Center be used? (This would include the use of the RedCap data base program)	Yes
2. Will the pediatric unit be used?	No
3. Will the adult unit be used?	No
4. In what capacity will the CTSC be used? Please explain	Core Lab (storage of specimens) and REDCAP.

Additional Form/s

Protocol Number: 1612017838 **Title:** Accessible Care Intervention for Engaging People who Inject Illicit Drugs (PWID) in Hepatitis C Care **Principinal Investigator:** Marks, Kristen

Questionnaire Name: Informed Consent for Prod

Question:	Answer:
1. Are you requesting a waiver of informed consent, either written or oral? If you require a waiver of informed consent for some, but not all subjects, please answer no to this question and indicate in the open text field which subjects you are requesting a waiver of informed consent from (e.g., you will be obtaining consent from some subjects, but others are lost to follow-up).	No
2. Are you obtaining written consent?	Yes
3. Describe procedures used to obtain informed consent (and minor assent, if applicable), including how and where informed consent (and assent, if applicable) will be obtained.	Informed consent will be obtained from all participants in the study at the respective syringe exchange facility (Lower East Side Harm Reduction Center). Consent will be obtained by a research staff member within a private office located in the syringe exchange facility. Participants who are unaware of their hepatitis C infection status will first be consented with the 'Screening' consent form. All individuals who are hepatitis C infected, and thus eligible for the full accessible care study, will be consented with the full paper consent form.
4. Describe what measures will be taken to ensure subjects' understanding of the protocol is consistent with the protocol as written.	Participants will be read the consent form by a member of the research staff. Throughout the reading of the form, and after, participants will have an opportunity to ask for clarification of the document. At the end of the reading participants will be ask if they have any questions. A copy of the sign consent form will be provided to the participant, and they will be encouraged to seek out a member of the study staff if they have further questions at whatever time in the future.
5. Will this study require the use of a legally authorized representative (LAR) to be utilized during the consenting process?	No
6. Do you expect to enroll non-English speaking subjects?	No

Additional Form/s

**Protocol Number:**	1612017838	**Title:**	Accessible Care Intervention for Engaging People who Inject Illicit Drugs (PWID) in Hepatitis C Care	**Prinicipal Investigator:**	Marks, Kristen

Questionnaire Name: Medical Devices Questionnaire

**Question:**	**Answer:**
1. Will any medical devices as defined by FDA regulations be used in this protocol?	No
