# Supplementary material for: Reduced injection risk behavior with co-located hepatitis C treatment at a syringe service program: The accessible care model
Source: PLoS One. 2024 Aug 29;19(8):e0308102. doi: 10.1371/journal.pone.0308102 (PMC11361571; doi:10.1371/journal.pone.0308102)
Supplement: S2 File — (HTM) [file pone.0308102.s004.htm]

SAS Output


|  |
| --- |
| In the past 30 days, how many days have you injected drugs? |

  

The GLIMMIX Procedure

| Model Information | |
| --- | --- |
| Data Set | BE.ACTRANSPOSE20231112 |
| Response Variable | InjB\_Inj\_30InjYN\_catg |
| Response Distribution | Multinomial (ordered) |
| Link Function | Cumulative Logit |
| Variance Function | Default |
| Variance Matrix Blocked By | subject |
| Estimation Technique | Maximum Likelihood |
| Likelihood Approximation | Gauss-Hermite Quadrature |
| Degrees of Freedom Method | Containment |

  

|  |  |
| --- | --- |
| Number of Observations Read | 611 |
| Number of Observations Used | 611 |

  

| Response Profile | | |
| --- | --- | --- |
| Ordered Value | InjB\_Inj\_30InjYN\_catg | Total Frequency |
| 1 | 3 | 167 |
| 2 | 2 | 78 |
| 3 | 1 | 131 |
| 4 | 0 | 235 |
| The GLIMMIX procedure is modeling the probabilities of levels of InjB\_Inj\_30InjYN\_catg having lower Ordered Values in the Response Profile table. | | |
| --- | --- | --- |

  

| Dimensions | |
| --- | --- |
| G-side Cov. Parameters | 1 |
| Columns in X | 14 |
| Columns in Z per Subject | 1 |
| Subjects (Blocks in V) | 165 |
| Max Obs per Subject | 5 |

  

| Optimization Information | |
| --- | --- |
| Optimization Technique | Dual Quasi-Newton |
| Parameters in Optimization | 9 |
| Lower Boundaries | 1 |
| Upper Boundaries | 0 |
| Fixed Effects | Not Profiled |
| Starting From | GLM estimates |
| Quadrature Points | 15 |

  

|  |
| --- |
| Convergence criterion (GCONV=1E-8) satisfied. |

  

| Fit Statistics | |
| --- | --- |
| -2 Log Likelihood | 1437.76 |
| AIC (smaller is better) | 1455.76 |
| AICC (smaller is better) | 1456.06 |
| BIC (smaller is better) | 1483.71 |
| CAIC (smaller is better) | 1492.71 |
| HQIC (smaller is better) | 1467.11 |

  

| Fit Statistics for Conditional Distribution | |
| --- | --- |
| -2 log L(InjB\_Inj\_30InjYN\_catg | r. effects) | 1151.51 |

  

| Covariance Parameter Estimates | | | | |
| --- | --- | --- | --- | --- |
| Cov Parm | Subject | Estimate | Standard Error | Gradient |
| UN(1,1) | subject | 2.5365 | 0.5600 | -0.00002 |

  

| Solutions for Fixed Effects | | | | | | | | | |
| --- | --- | --- | --- | --- | --- | --- | --- | --- | --- |
| Effect | bl: In the past 30 days, how many days have you injected drugs? | ac | Cascade4\_tx\_initiated1 | Estimate | Standard Error | DF | t Value | Pr > |t| | Gradient |
| Intercept | 3 |  |  | -0.05451 | 0.3436 | 161 | -0.16 | 0.8741 | -0.00243 |
| Intercept | 2 |  |  | 0.8371 | 0.3458 | 161 | 2.42 | 0.0166 | 0.000694 |
| Intercept | 1 |  |  | 2.2027 | 0.3609 | 161 | 6.10 | <.0001 | 0.001438 |
| ac |  | 1 |  | 1.3163 | 0.6587 | 442 | 2.00 | 0.0463 | -0.00027 |
| ac |  | 0 |  | 0 | . | . | . | . | . |
| time |  |  |  | -0.2770 | 0.08222 | 442 | -3.37 | 0.0008 | -0.00114 |
| time\*ac |  | 1 |  | -0.2499 | 0.1161 | 442 | -2.15 | 0.0318 | -0.00062 |
| time\*ac |  | 0 |  | 0 | . | . | . | . | . |
| Cascade4\_tx\_initiate |  |  | 1 | -1.2151 | 0.4830 | 442 | -2.52 | 0.0122 | -0.00043 |
| Cascade4\_tx\_initiate |  |  | 0 | 0 | . | . | . | . | . |
| ac\*Cascade4\_tx\_initi |  | 1 | 1 | -0.4310 | 0.7520 | 442 | -0.57 | 0.5668 | -0.00037 |
| ac\*Cascade4\_tx\_initi |  | 1 | 0 | 0 | . | . | . | . | . |
| ac\*Cascade4\_tx\_initi |  | 0 | 1 | 0 | . | . | . | . | . |
| ac\*Cascade4\_tx\_initi |  | 0 | 0 | 0 | . | . | . | . | . |

  

| Type III Tests of Fixed Effects | | | | |
| --- | --- | --- | --- | --- |
| Effect | Num DF | Den DF | F Value | Pr > F |
| ac | 1 | 442 | 5.15 | 0.0238 |
| time | 1 | 442 | 44.99 | <.0001 |
| time\*ac | 1 | 442 | 4.64 | 0.0318 |
| Cascade4\_tx\_initiate | 1 | 442 | 14.16 | 0.0002 |
| ac\*Cascade4\_tx\_initi | 1 | 442 | 0.33 | 0.5668 |

  

| Tests of Covariance Parameters Based on the Likelihood | | | | | |
| --- | --- | --- | --- | --- | --- |
| Label | DF | -2 Log Like | ChiSq | Pr > ChiSq | Note |
| Need Random Intercept? | 1 | 1535.98 | 98.22 | <.0001 | MI |

  

|  |
| --- |
| MI: P-value based on a mixture of chi-squares. |

---


|  |
| --- |
| How many injection events over the last 30 days? |

  

The GLIMMIX Procedure

| Model Information | |
| --- | --- |
| Data Set | BE.ACTRANSPOSE20231112 |
| Response Variable | Inj\_30InjYN\_InjXDay\_catg |
| Response Distribution | Multinomial (ordered) |
| Link Function | Cumulative Logit |
| Variance Function | Default |
| Variance Matrix Blocked By | subject |
| Estimation Technique | Maximum Likelihood |
| Likelihood Approximation | Gauss-Hermite Quadrature |
| Degrees of Freedom Method | Containment |

  

|  |  |
| --- | --- |
| Number of Observations Read | 611 |
| Number of Observations Used | 611 |

  

| Response Profile | | |
| --- | --- | --- |
| Ordered Value | Inj\_30InjYN\_InjXDay\_catg | Total Frequency |
| 1 | 3 | 125 |
| 2 | 2 | 98 |
| 3 | 1 | 152 |
| 4 | 0 | 236 |
| The GLIMMIX procedure is modeling the probabilities of levels of Inj\_30InjYN\_InjXDay\_catg having lower Ordered Values in the Response Profile table. | | |
| --- | --- | --- |

  

| Dimensions | |
| --- | --- |
| G-side Cov. Parameters | 1 |
| Columns in X | 14 |
| Columns in Z per Subject | 1 |
| Subjects (Blocks in V) | 165 |
| Max Obs per Subject | 5 |

  

| Optimization Information | |
| --- | --- |
| Optimization Technique | Dual Quasi-Newton |
| Parameters in Optimization | 9 |
| Lower Boundaries | 1 |
| Upper Boundaries | 0 |
| Fixed Effects | Not Profiled |
| Starting From | GLM estimates |
| Quadrature Points | 15 |

  

|  |
| --- |
| Convergence criterion (GCONV=1E-8) satisfied. |

  

| Fit Statistics | |
| --- | --- |
| -2 Log Likelihood | 1435.69 |
| AIC (smaller is better) | 1453.69 |
| AICC (smaller is better) | 1453.98 |
| BIC (smaller is better) | 1481.64 |
| CAIC (smaller is better) | 1490.64 |
| HQIC (smaller is better) | 1465.03 |

  

| Fit Statistics for Conditional Distribution | |
| --- | --- |
| -2 log L(Inj\_30InjYN\_InjXDay\_catg | r. effects) | 1131.20 |

  

| Covariance Parameter Estimates | | | | |
| --- | --- | --- | --- | --- |
| Cov Parm | Subject | Estimate | Standard Error | Gradient |
| UN(1,1) | subject | 2.7651 | 0.5884 | -0.00114 |

  

| Solutions for Fixed Effects | | | | | | | | | |
| --- | --- | --- | --- | --- | --- | --- | --- | --- | --- |
| Effect | bl: How many times did you inject over the last 30 days? [NEW VARIABLE - if they didn't inject should be zero] (#times/day, topcoded at 10). | ac | Cascade4\_tx\_initiated1 | Estimate | Standard Error | DF | t Value | Pr > |t| | Gradient |
| Intercept | 3 |  |  | -0.7584 | 0.3449 | 161 | -2.20 | 0.0293 | 0.001996 |
| Intercept | 2 |  |  | 0.5279 | 0.3424 | 161 | 1.54 | 0.1251 | 0.000864 |
| Intercept | 1 |  |  | 2.1802 | 0.3588 | 161 | 6.08 | <.0001 | -0.00094 |
| ac |  | 1 |  | 1.7927 | 0.6631 | 442 | 2.70 | 0.0071 | 0.000295 |
| ac |  | 0 |  | 0 | . | . | . | . | . |
| time |  |  |  | -0.2789 | 0.08076 | 442 | -3.45 | 0.0006 | -0.00132 |
| time\*ac |  | 1 |  | -0.3571 | 0.1165 | 442 | -3.06 | 0.0023 | -0.00195 |
| time\*ac |  | 0 |  | 0 | . | . | . | . | . |
| Cascade4\_tx\_initiate |  |  | 1 | -1.2362 | 0.4946 | 442 | -2.50 | 0.0128 | -0.00074 |
| Cascade4\_tx\_initiate |  |  | 0 | 0 | . | . | . | . | . |
| ac\*Cascade4\_tx\_initi |  | 1 | 1 | -0.4885 | 0.7670 | 442 | -0.64 | 0.5245 | -0.00062 |
| ac\*Cascade4\_tx\_initi |  | 1 | 0 | 0 | . | . | . | . | . |
| ac\*Cascade4\_tx\_initi |  | 0 | 1 | 0 | . | . | . | . | . |
| ac\*Cascade4\_tx\_initi |  | 0 | 0 | 0 | . | . | . | . | . |

  

| Type III Tests of Fixed Effects | | | | |
| --- | --- | --- | --- | --- |
| Effect | Num DF | Den DF | F Value | Pr > F |
| ac | 1 | 442 | 9.95 | 0.0017 |
| time | 1 | 442 | 56.81 | <.0001 |
| time\*ac | 1 | 442 | 9.39 | 0.0023 |
| Cascade4\_tx\_initiate | 1 | 442 | 14.53 | 0.0002 |
| ac\*Cascade4\_tx\_initi | 1 | 442 | 0.41 | 0.5245 |

  

| Tests of Covariance Parameters Based on the Likelihood | | | | | |
| --- | --- | --- | --- | --- | --- |
| Label | DF | -2 Log Like | ChiSq | Pr > ChiSq | Note |
| Need Random Intercept? | 1 | 1545.08 | 109.40 | <.0001 | MI |

  

|  |
| --- |
| MI: P-value based on a mixture of chi-squares. |

---


|  |
| --- |
| No. of times using previously used syringes in the last 30 days |

  

The GLIMMIX Procedure

| Model Information | |
| --- | --- |
| Data Set | BE.ACTRANSPOSE20230216A |
| Response Variable | InjB\_Inj\_30XUseRecSyr\_catg |
| Response Distribution | Multinomial (ordered) |
| Link Function | Cumulative Logit |
| Variance Function | Default |
| Variance Matrix Blocked By | subject |
| Estimation Technique | Maximum Likelihood |
| Likelihood Approximation | Gauss-Hermite Quadrature |
| Degrees of Freedom Method | Containment |

  

|  |  |
| --- | --- |
| Number of Observations Read | 622 |
| Number of Observations Used | 621 |

  

| Response Profile | | |
| --- | --- | --- |
| Ordered Value | InjB\_Inj\_30XUseRecSyr\_catg | Total Frequency |
| 1 | 2 | 4 |
| 2 | 1 | 27 |
| 3 | 0 | 590 |
| The GLIMMIX procedure is modeling the probabilities of levels of InjB\_Inj\_30XUseRecSyr\_catg having lower Ordered Values in the Response Profile table. | | |
| --- | --- | --- |

  

| Dimensions | |
| --- | --- |
| G-side Cov. Parameters | 1 |
| Columns in X | 13 |
| Columns in Z per Subject | 1 |
| Subjects (Blocks in V) | 165 |
| Max Obs per Subject | 5 |

  

| Optimization Information | |
| --- | --- |
| Optimization Technique | Dual Quasi-Newton |
| Parameters in Optimization | 8 |
| Lower Boundaries | 1 |
| Upper Boundaries | 0 |
| Fixed Effects | Not Profiled |
| Starting From | GLM estimates |
| Quadrature Points | 15 |

  

|  |
| --- |
| Convergence criterion (GCONV=1E-8) satisfied. |

  

| Fit Statistics | |
| --- | --- |
| -2 Log Likelihood | 250.30 |
| AIC (smaller is better) | 266.30 |
| AICC (smaller is better) | 266.54 |
| BIC (smaller is better) | 291.15 |
| CAIC (smaller is better) | 299.15 |
| HQIC (smaller is better) | 276.39 |

  

| Fit Statistics for Conditional Distribution | |
| --- | --- |
| -2 log L(InjB\_Inj\_30XUseRecSyr\_catg | r. effects) | 187.57 |

  

| Covariance Parameter Estimates | | | | |
| --- | --- | --- | --- | --- |
| Cov Parm | Subject | Estimate | Standard Error | Gradient |
| UN(1,1) | subject | 1.5961 | 1.0723 | 8.452E-7 |

  

| Solutions for Fixed Effects | | | | | | | | | |
| --- | --- | --- | --- | --- | --- | --- | --- | --- | --- |
| Effect | InjB\_Inj\_30XUseRecSyr\_catg | ac | Cascade4\_tx\_initiated1 | Estimate | Standard Error | DF | t Value | Pr > |t| | Gradient |
| Intercept | 2 |  |  | -4.8862 | 0.8970 | 161 | -5.45 | <.0001 | -8.39E-6 |
| Intercept | 1 |  |  | -2.6099 | 0.7135 | 161 | -3.66 | 0.0003 | 0.000016 |
| ac |  | 1 |  | 0.9779 | 1.0478 | 453 | 0.93 | 0.3511 | 0.000012 |
| ac |  | 0 |  | 0 | . | . | . | . | . |
| time |  |  |  | -0.4219 | 0.2289 | 453 | -1.84 | 0.0659 | 0.000024 |
| time\*ac |  | 1 |  | -0.2403 | 0.3186 | 453 | -0.75 | 0.4510 | 0.000019 |
| time\*ac |  | 0 |  | 0 | . | . | . | . | . |
| Cascade4\_tx\_initiate |  |  | 1 | -0.4432 | 0.8097 | 453 | -0.55 | 0.5844 | 0.000017 |
| Cascade4\_tx\_initiate |  |  | 0 | 0 | . | . | . | . | . |
| ac\*Cascade4\_tx\_initi |  | 1 | 1 | 0.1659 | 1.1486 | 453 | 0.14 | 0.8852 | 0.000016 |
| ac\*Cascade4\_tx\_initi |  | 1 | 0 | 0 | . | . | . | . | . |
| ac\*Cascade4\_tx\_initi |  | 0 | 1 | 0 | . | . | . | . | . |
| ac\*Cascade4\_tx\_initi |  | 0 | 0 | 0 | . | . | . | . | . |

  

| Type III Tests of Fixed Effects | | | | |
| --- | --- | --- | --- | --- |
| Effect | Num DF | Den DF | F Value | Pr > F |
| ac | 1 | 453 | 1.49 | 0.2227 |
| time | 1 | 453 | 11.12 | 0.0009 |
| time\*ac | 1 | 453 | 0.57 | 0.4510 |
| Cascade4\_tx\_initiate | 1 | 453 | 0.39 | 0.5303 |
| ac\*Cascade4\_tx\_initi | 1 | 453 | 0.02 | 0.8852 |

  

| Tests of Covariance Parameters Based on the Likelihood | | | | | |
| --- | --- | --- | --- | --- | --- |
| Label | DF | -2 Log Like | ChiSq | Pr > ChiSq | Note |
| Need Random Intercept? | 1 | 254.89 | 4.59 | 0.0161 | MI |

  

|  |
| --- |
| MI: P-value based on a mixture of chi-squares. |

---


|  |
| --- |
| No. of times of sharing cookers in the last 30 days |

  

The GLIMMIX Procedure

| Model Information | |
| --- | --- |
| Data Set | BE.ACTRANSPOSE20230216A |
| Response Variable | InjB\_Inj\_30XShareCooker\_catg |
| Response Distribution | Multinomial (ordered) |
| Link Function | Cumulative Logit |
| Variance Function | Default |
| Variance Matrix Blocked By | subject |
| Estimation Technique | Maximum Likelihood |
| Likelihood Approximation | Gauss-Hermite Quadrature |
| Degrees of Freedom Method | Containment |

  

|  |  |
| --- | --- |
| Number of Observations Read | 622 |
| Number of Observations Used | 621 |

  

| Response Profile | | |
| --- | --- | --- |
| Ordered Value | InjB\_Inj\_30XShareCooker\_catg | Total Frequency |
| 1 | 3 | 14 |
| 2 | 2 | 20 |
| 3 | 1 | 72 |
| 4 | 0 | 515 |
| The GLIMMIX procedure is modeling the probabilities of levels of InjB\_Inj\_30XShareCooker\_catg having lower Ordered Values in the Response Profile table. | | |
| --- | --- | --- |

  

| Dimensions | |
| --- | --- |
| G-side Cov. Parameters | 1 |
| Columns in X | 14 |
| Columns in Z per Subject | 1 |
| Subjects (Blocks in V) | 165 |
| Max Obs per Subject | 5 |

  

| Optimization Information | |
| --- | --- |
| Optimization Technique | Dual Quasi-Newton |
| Parameters in Optimization | 9 |
| Lower Boundaries | 1 |
| Upper Boundaries | 0 |
| Fixed Effects | Not Profiled |
| Starting From | GLM estimates |
| Quadrature Points | 15 |

  

|  |
| --- |
| Convergence criterion (GCONV=1E-8) satisfied. |

  

| Fit Statistics | |
| --- | --- |
| -2 Log Likelihood | 660.83 |
| AIC (smaller is better) | 678.83 |
| AICC (smaller is better) | 679.13 |
| BIC (smaller is better) | 706.79 |
| CAIC (smaller is better) | 715.79 |
| HQIC (smaller is better) | 690.18 |

  

| Fit Statistics for Conditional Distribution | |
| --- | --- |
| -2 log L(InjB\_Inj\_30XShareCooker\_catg | r. effects) | 462.23 |

  

| Covariance Parameter Estimates | | | | |
| --- | --- | --- | --- | --- |
| Cov Parm | Subject | Estimate | Standard Error | Gradient |
| UN(1,1) | subject | 3.5948 | 1.0999 | 1.084E-6 |

  

| Solutions for Fixed Effects | | | | | | | | | |
| --- | --- | --- | --- | --- | --- | --- | --- | --- | --- |
| Effect | InjB\_Inj\_30XShareCooker\_catg | ac | Cascade4\_tx\_initiated1 | Estimate | Standard Error | DF | t Value | Pr > |t| | Gradient |
| Intercept | 3 |  |  | -4.1115 | 0.5510 | 161 | -7.46 | <.0001 | 4.047E-6 |
| Intercept | 2 |  |  | -2.9377 | 0.4914 | 161 | -5.98 | <.0001 | -2.68E-6 |
| Intercept | 1 |  |  | -1.1331 | 0.4471 | 161 | -2.53 | 0.0122 | 5.96E-6 |
| ac |  | 1 |  | 0.5424 | 0.8315 | 452 | 0.65 | 0.5145 | 4.538E-6 |
| ac |  | 0 |  | 0 | . | . | . | . | . |
| time |  |  |  | -0.1269 | 0.1108 | 452 | -1.14 | 0.2529 | 0.000019 |
| time\*ac |  | 1 |  | -0.3901 | 0.1838 | 452 | -2.12 | 0.0344 | 0.00001 |
| time\*ac |  | 0 |  | 0 | . | . | . | . | . |
| Cascade4\_tx\_initiate |  |  | 1 | -1.3692 | 0.6750 | 452 | -2.03 | 0.0431 | 4.448E-6 |
| Cascade4\_tx\_initiate |  |  | 0 | 0 | . | . | . | . | . |
| ac\*Cascade4\_tx\_initi |  | 1 | 1 | 0.09788 | 1.0014 | 452 | 0.10 | 0.9222 | 3.237E-6 |
| ac\*Cascade4\_tx\_initi |  | 1 | 0 | 0 | . | . | . | . | . |
| ac\*Cascade4\_tx\_initi |  | 0 | 1 | 0 | . | . | . | . | . |
| ac\*Cascade4\_tx\_initi |  | 0 | 0 | 0 | . | . | . | . | . |

  

| Type III Tests of Fixed Effects | | | | |
| --- | --- | --- | --- | --- |
| Effect | Num DF | Den DF | F Value | Pr > F |
| ac | 1 | 452 | 0.80 | 0.3702 |
| time | 1 | 452 | 12.04 | 0.0006 |
| time\*ac | 1 | 452 | 4.50 | 0.0344 |
| Cascade4\_tx\_initiate | 1 | 452 | 6.66 | 0.0102 |
| ac\*Cascade4\_tx\_initi | 1 | 452 | 0.01 | 0.9222 |

  

| Tests of Covariance Parameters Based on the Likelihood | | | | | |
| --- | --- | --- | --- | --- | --- |
| Label | DF | -2 Log Like | ChiSq | Pr > ChiSq | Note |
| Need Random Intercept? | 1 | 715.26 | 54.42 | <.0001 | MI |

  

|  |
| --- |
| MI: P-value based on a mixture of chi-squares. |

---


|  |
| --- |
| treatment for opioid use disorder in the last 90 days |

  

The GLIMMIX Procedure

| Model Information | |
| --- | --- |
| Data Set | BE.ACTRANSPOSE20230216A |
| Response Variable | DrugTxB\_DrugTx\_DrugTx90YN |
| Response Distribution | Multinomial (ordered) |
| Link Function | Cumulative Logit |
| Variance Function | Default |
| Variance Matrix Blocked By | subject |
| Estimation Technique | Maximum Likelihood |
| Likelihood Approximation | Gauss-Hermite Quadrature |
| Degrees of Freedom Method | Containment |

  

|  |  |
| --- | --- |
| Number of Observations Read | 622 |
| Number of Observations Used | 622 |

  

| Response Profile | | |
| --- | --- | --- |
| Ordered Value | DrugTxB\_DrugTx\_DrugTx90YN | Total Frequency |
| 1 | 0 | 204 |
| 2 | 1 | 418 |
| The GLIMMIX procedure is modeling the probabilities of levels of DrugTxB\_DrugTx\_DrugTx90YN having lower Ordered Values in the Response Profile table. | | |
| --- | --- | --- |

  

| Dimensions | |
| --- | --- |
| G-side Cov. Parameters | 1 |
| Columns in X | 12 |
| Columns in Z per Subject | 1 |
| Subjects (Blocks in V) | 165 |
| Max Obs per Subject | 5 |

  

| Optimization Information | |
| --- | --- |
| Optimization Technique | Dual Quasi-Newton |
| Parameters in Optimization | 7 |
| Lower Boundaries | 1 |
| Upper Boundaries | 0 |
| Fixed Effects | Not Profiled |
| Starting From | GLM estimates |
| Quadrature Points | 15 |

  

|  |
| --- |
| Convergence criterion (GCONV=1E-8) satisfied. |

  

| Fit Statistics | |
| --- | --- |
| -2 Log Likelihood | 649.17 |
| AIC (smaller is better) | 663.17 |
| AICC (smaller is better) | 663.35 |
| BIC (smaller is better) | 684.91 |
| CAIC (smaller is better) | 691.91 |
| HQIC (smaller is better) | 671.99 |

  

| Fit Statistics for Conditional Distribution | |
| --- | --- |
| -2 log L(DrugTxB\_DrugTx\_DrugTx90YN | r. effects) | 382.13 |

  

| Covariance Parameter Estimates | | | | |
| --- | --- | --- | --- | --- |
| Cov Parm | Subject | Estimate | Standard Error | Gradient |
| UN(1,1) | subject | 5.3969 | 1.4240 | 0.000063 |

  

| Solutions for Fixed Effects | | | | | | | | | |
| --- | --- | --- | --- | --- | --- | --- | --- | --- | --- |
| Effect | DrugTxB\_DrugTx\_DrugTx90YN | ac | Cascade4\_tx\_initiated1 | Estimate | Standard Error | DF | t Value | Pr > |t| | Gradient |
| Intercept | 0 |  |  | -2.2621 | 0.5206 | 161 | -4.35 | <.0001 | -0.00017 |
| ac |  | 1 |  | -0.01183 | 0.9484 | 455 | -0.01 | 0.9901 | 0.000601 |
| ac |  | 0 |  | 0 | . | . | . | . | . |
| time |  |  |  | 0.5234 | 0.1219 | 455 | 4.29 | <.0001 | -0.00254 |
| time\*ac |  | 1 |  | -0.1848 | 0.1649 | 455 | -1.12 | 0.2632 | 0.001712 |
| time\*ac |  | 0 |  | 0 | . | . | . | . | . |
| Cascade4\_tx\_initiate |  |  | 1 | -0.8335 | 0.7210 | 455 | -1.16 | 0.2483 | 0.000299 |
| Cascade4\_tx\_initiate |  |  | 0 | 0 | . | . | . | . | . |
| ac\*Cascade4\_tx\_initi |  | 1 | 1 | 0.5006 | 1.1080 | 455 | 0.45 | 0.6516 | 0.000522 |
| ac\*Cascade4\_tx\_initi |  | 1 | 0 | 0 | . | . | . | . | . |
| ac\*Cascade4\_tx\_initi |  | 0 | 1 | 0 | . | . | . | . | . |
| ac\*Cascade4\_tx\_initi |  | 0 | 0 | 0 | . | . | . | . | . |

  

| Type III Tests of Fixed Effects | | | | |
| --- | --- | --- | --- | --- |
| Effect | Num DF | Den DF | F Value | Pr > F |
| ac | 1 | 455 | 0.11 | 0.7442 |
| time | 1 | 455 | 24.71 | <.0001 |
| time\*ac | 1 | 455 | 1.26 | 0.2632 |
| Cascade4\_tx\_initiate | 1 | 455 | 1.11 | 0.2930 |
| ac\*Cascade4\_tx\_initi | 1 | 455 | 0.20 | 0.6516 |

  

| Tests of Covariance Parameters Based on the Likelihood | | | | | |
| --- | --- | --- | --- | --- | --- |
| Label | DF | -2 Log Like | ChiSq | Pr > ChiSq | Note |
| Need Random Intercept? | 1 | 767.41 | 118.24 | <.0001 | MI |

  

|  |
| --- |
| MI: P-value based on a mixture of chi-squares. |
