# Supplementary material for: Reduced injection risk behavior with co-located hepatitis C treatment at a syringe service program: The accessible care model
Source: PLoS One. 2024 Aug 29;19(8):e0308102. doi: 10.1371/journal.pone.0308102 (PMC11361571; doi:10.1371/journal.pone.0308102)
Supplement: Attachment — Submitted filename: Response to Reviewers.docx [file pone.0308102.s005.docx]

Thank you for reviewing our manuscript and for providing your feedback– helping make our manuscript stronger. Below are the reviewers comments (in bold) and our responses (in plain font). Edited text is shown in italic font.

**Reviewer 1**

1. **Page 3, lines 48–51: I suggest considering reporting coefficients of interactions in a clinical term. In addition, I suggest reporting 95% confidence intervals.**

- We are now reporting our results in odds ratios instead of beta-coefficients. We hope that this makes our results easier to understand and apply clinically. We have described the interpretation of our results in the following sentences:

*The AC-by-time effect on the number of injection days in the past 30 days had an OR of 0.78 (95% CI= 0.62-0.98), demonstrating significantly decreased odds of higher rates of injection days in the AC arm compared to the UC arm for each one unit increase in point of time (Table 3). This indicates a higher likelihood of an individual from the AC group to move to a category of a lower range of injection days over time (Figure 1). Similar results were seen in the number of injection events with AC-by-time effect with OR of 0.70 (95% CI=0.56-0.88), indicating a higher likelihood of an AC participant being in a category of lower injection event frequencies over time (Figure 2).* (Line 269-276)

- 95% confidence intervals have been added.

2. **Page 9, lines 190–191: I suggest adding ‘mean with standard deviation’ per tables.**

Sentence has been reworded to include “mean with standard deviation”:

*The baseline characteristic of participants were reported as frequencies with percentages and mean values with standard deviations.* (Line 190-191)

3. **Page 9, lines 191–192: These tests were not reported. I suggest excluding this sentence.**

Tests not reported/irrelevant have been excluded.

4. **Page 9, lines 194–195: I suggest clarifying the calling out of Table 2. The title of Table 2 indicated “baseline.” However, the sentence described the ordinal category at all study points.**

Sentence has been changed to clarify the calling out of table 2:

*At baseline (Table 2) and at each study timepoint (Table 3), participants were grouped into ordinal categories of a range of number of injection events/days in the last 30 days based on their interview answers.* (Line 199-201)

5. **Page 9, lines 197–198: I recommend providing additional information about the generalized estimating equations (GEE) such as the distribution, the link function, and model specification. Also, please confirm the use of t-test in GEE. The most popular test in GEE is the Wald test with naive or robust standard errors.**

- The use of t-test has been confirmed (Line).
- We changed our statistical analysis from GEE to generalized linear mixed model (GLMM) to incorporate random intercept effect to improve the model
- Details about the distribution, link function, and the models have been included:

*-we modeled the data using generalized linear mixed model (GLMM) to evaluate the main effect of intervention, time, and the interaction effect of intervention and time. Additionally, initiation to treatment and its interaction with intervention were included as covariates, accounting for potential confounding effect among those who initiated treatment vs those who did not. Given our focus on testing for trends, time was treated as a numeric variable. For the treatment of opioid use disorder, since the variable is dichotomous, a binary distribution with logit link function was used. For injection frequency and equipment sharing frequency variables, a multinomial distribution with cumulative logit link function was used. All models included a random intercept.* (Line 211-219)

6. **Page 9, lines 198–200: I recommend assessing the balance in the numbers of days and events of injection drug use at baseline between arms and determining the need for adjustment based on this balance, rather than solely on statistical significance.**

Baseline characteristics, including number of days and events of injection use, were balanced between the AC and UC arms with the exception of the percentage of participants recently incarcerated being higher in the UC arm. The randomized controlled trial design of this study also ensured balance through randomization when assigning participants to the two arms.

**7. Page 10, Table 1: Were there any concerns regarding imbalance in the parent study?**

The only imbalance between the baseline characteristics of participants in the two arms was the percentage of recent incarceration being higher in the UC arm compared to the AC arm. We have added a footnote under Table 1 and a line in the results section highlighting this:

*^b^ Statistical difference between AC and UC with p<0.05* (Table 1)

*The baseline characteristics of the two arms were balanced except for the percentage of participants in the UC arm incarcerated in the last 90 (12.0%) being significantly higher than in the AC arm (2.4%).* (Line 246-248)

8. **Page 11, lines 220–221: Would it be possible to declare the balance without referring the statistical significance?**

Given that this study was a randomized controlled trial the design itself ensured balance through randomization. We referred to the lack of statistical significance to further emphasize this and also provide the raw data in table 2. The following sentences have also been added to imply balance by inspection:

*The majority of participants in both treatment arms were male (77.6%), with a mean age of 42.0. The AC group had more participants aged 45 or older than the UC group (45.1% vs. 36.1), while the UC group had more participants aged 31-44 (51.8% vs. 41.5%). There were slightly more Hispanic participants in the UC arm (62.7%) than in the AC arm (54.9%). The UC arm had 51.8% of participants report the use of heroin in the last 90 days compared to the 43.9% in the AC arm. In both groups, more than half of study participants experienced homelessness in the last 3 months. Most participants (84%) had a history of incarceration with 7.3% having been incarcerated within the last 90 days. Most participants (93.9%) were publicly insured. More participants in the AC arm had seen a non-HCV clinician in the last 90 days (53.7%) than participants in the UC arm (45.8%). The baseline characteristics of the two arms were balanced except for the percentage of participants in the UC arm incarcerated in the last 90 (12.0%) being significantly higher than in the AC arm (2.4%).* (Line 236-248)

**9. Page 12, lines 226–228: Can you please elaborate the average number of participants in non-attendance? It was not clear how to interpret the numbers of 53.5 and 59.**

If the number of non-attendances between the two arms across all 4 time points were taken and averaged, the resulting number is 53.5. The highest number of non-attendances (59 non-attendances) occurred at the 3 month mark. The previous sentence has been worded:

*An average of 53.5 participants were in non-attendance at each follow-up visit throughout the four timepoints. The highest number of non-attendance occurred at the 3-month follow-up with 59 participants absent across both groups.* (Line 264-266)

10. **Page 12, lines 228–229: I suggest considering removing this sentence.**

Given the theoretical possibility that there may be higher attendance rates at follow-up interviews in the AC group, we felt that it was important to explicitly state that there was no significant difference in the non-attendance rates between the AC and UC arms.

11. **Page 12, line 231: Did the intervention-by-time effect analysis refer to the GEE with interaction terms between intervention and time? If so, I suggest providing the specifications in the Statistical Analysis section. See Comment #5 above.**

We are now using GLMM instead of GEE. Specifications have been provided in the statistical analysis section. Please see our response to comment #5 above.

12. **Page 12, lines 233–239: Would you mind providing the SPSS outputs from GEE as a supplementary material or an attachment to the response? It was not easy to envision the models.**

We have changed our model to the GLMM model. We have provided the SAS output as a supplementary material.

13. **Page 13, Table 3: The coefficient of the interaction would only be directly interpretable if the outcome was continuous; i.e., the distribution of GEE was Gaussian and the link function was identity. Modeling the percentage as a continuous outcome might not be ideal but it was acceptable given GEE.**

We have changed the manuscript to report odds ratios using GLMM instead of beta-coefficient values. Please see our response to comment #5 above.

**Reviewer 2**

1. **This paper describes a secondary analysis of a previously published paper and rehashes those findings at length rather than referring the reader to the publication and summarizing the results here in brief.**

We shortened the description and removed the results of the primary outcomes of the parent study in response to this comment. The retained details of the parent study were felt to be relevant to the outcomes of the current secondary analysis.

*Deleted:*

- The AC model provides low-threshold HCV treatment co-located at a syringe service program (SSP); involving proactive engagement with patients, flexible appointments, assistance in obtaining coverage for the cost of DAA agents, and a non-judgmental atmosphere which mitigates previously stigmatizing encounters PWID have experienced in healthcare settings.^19^
- The primary outcome of the parent Accessible Care study was the percentage participants achieving SVR12 within 12 months of study enrollment, which demonstrated a significantly higher SVR12 rate in the AC versus UC arm (67.1% vs. 22.9%, p<0.001).^9^

2. **Moreover, in the Discussion, the authors describe the key outcome differences in the parent study due to "...rates of advancement along the care cascade" (p. 18/lines 275-78), which is not mentioned in the previous description of the primary study.**

We have reworded the sentence to include this in the introduction where we are outlining the findings of the parent study and its relevance to the current secondary analysis:

*The AC arm demonstrated significantly higher sustained virologic response (SVR) rates compared to the UC arm from higher rates of advancement along the care cascade in the AC arm . Successful treatment itself, in addition to a supportive care model, can be motivating for healthier lifestyles and choices.(9,20)* (Line 110-113)

3. **There is no mention of possible recall bias or acceptability bias despite the fact that participants were asked to enumerate the frequency of specific drug use behaviors over a 30-day period.**

We have included the following in the limitations section:

*Given that the frequency of injection and high-risk behavior frequencies were self-reported during interviews, the possibility of recall bias and social desirability bias is present. In this study, questionnaires were administered by interviewers. Assuming that individuals in the AC arm had stronger rapport with the care team, it is important to consider whether this led to underreporting due to concerns of disappointing the interviewer or if it led to more transparency.* (Lines 409-414)

4. **In addition, there are a number of awkward phrasings (e.g. "within PWID" rather than "among PWID") that could benefit from a less stilted style.**

We have gone through the manuscript and have reworded awkwardly phrased sentences.

5. **The tables are long (esp Table 3) and I wonder if there is a more visual way to display this information.**

Table 3 has been edited to be more succinct, have less white space, and present the data more clearly.

**Reviewer #3**

**This paper reports on the outcomes of a randomized clinical trial of an accessible care approach to providing Hepatitis C treatment relative to treatment as usual condition on the primary outcomes of frequency of injection drug use and engagement in high-risk injection drug use engagement. Results indicate that those randomized to the accessible care condition had lower frequency of injection drug use and high-risk injection practices relative to the treatment as usual condition.

The following are suggestions to improve the manuscript.**

1. **In the introduction (pg. 5): It would be helpful to clarify what patient navigation in the control arm entailed.**

We reworded the introduction to clarify the difference between the AC arm and the UC arm (primarily referral based and much more heavily relied on patient navigation):

*We previously reported results of a randomized controlled trial of the Accessible Care (AC) model which compared a supportive and on-site low-threshold treatment model (AC) to a referral and patient navigation-based model (usual care). (9,18)* (Line 107-109)

We also revised the description of the UC arm in the methods section to further highlight the difference between the two arms:

*Participants in the UC arm were connected to an on-site HCV patient navigator who was funded through the New York City Department of Health and part of the Check Hep C program. The patient navigator referred participants to HCV specialists at external hospitals/clinics for treatment and harm reduction programs. Similar to the AC arm’s care coordinator, the patient navigator also provided social support and assistance with insurance navigation. Contrastingly, support for treatment initiation and completion was not provided to the UC group.* (Line 158-163)

2. **The authors note in the introduction that past, previously reported results of this trial indicated the accessible care approach was associated with higher sustained virologic response relative to the usual care arm. The authors should make more explicit in the abstract that the primary outcome of the trial was not the primary outcome(s) being reported in this paper (i.e., injection drug use frequency, frequency of high-risk injection practices).**

We have revised the last paragraph of the Introduction to make more explicit the primary outcome of the current secondary analysis:

*To assess the changes in injection behaviors and practices of PWID associated with HCV treatment we analyzed data from the Accessible Care study.(6) The focus of this paper is to assess and compare the changes in injection behaviors, including injection frequency and frequency of high-risk injection behaviors, between PWID who received the AC intervention and usual care (UC) intervention.* (Line 115-119)

We have also edited the Methods section to re-emphasize the primary outcomes of this study:

*The primary outcomes of this paper were changes in the frequency of high-risk injection behaviors (sharing of injection equipment and use of previously used syringes), injection frequency (number of days of injection and total number of injection events in the last 30 days), and uptake of MOUD at 3, 6, 9, and 12 months relative to baseline.* (Line 181-185)

3. **In the methods, I would recommend describing the extent to which content focused on altering injection practices was included in either the accessible care or usual care conditions. I would assume this counseling is part of all the SSP services provided, but wasn’t clear whether there was additional emphasis provided on reducing frequency of injection drug use in either condition.**

Participants in the AC arm receiving DAA therapy were offered an on-site, interactive, oral presentation on re-infection prevention. Participants in the UC arm were not offered any on-site sessions. We have revised the methods section to make this difference clear:

*Exclusive to the AC arm, all participants receiving DAA therapy were offered an on-site and interactive re-infection prevention oral presentation adapted from the validated Staying Safe Intervention program.* (Line 153-155)

*The patient navigator referred participants to HCV specialists at external hospitals/clinics for treatment. Similar to the AC arm’s care coordinator, the patient navigator also provided social support and assistance with insurance navigation. Contrastingly, support for treatment initiation and completion was not provided to the UC group.* *There was no on-site education regarding re-infection prevention offered.* (Line 159-164)

4. **On page 9, the authors also indicate that uptake of MOUD is an outcome, but this hasn’t been addressed as an outcome in the manuscript to this point. There is a need for incorporating MOUD into the abstract and introduction.**

The last sentence of the methods section of the abstract was revised to mention MOUD:

*Participants attended follow-up appointments at 3, 6, 9, and 12 months during which they answered questions regarding injection drug use frequency, behaviors, and treatment for opioid use disorder.* (Line 36-38)

The following was added to the Introduction:

*As for harm reduction through medications for opioid use disorders (MOUD), the current literature highlights low retention rates in treatment programs.* (Line 99-101)

5. **Additional description of the measurement of the manuscript’s primary outcomes is needed. Further, how were categories of number of days injecting determined? What was a continuous measure not employed? The rationale for separating 29 days from 30 days is unclear, for example.**

- Method section describes the interviews conducted at each follow-up time point to collect data for the primary outcomes
- The following has been added to the Methods section to clarify how the ordinal categories were determined and our rationale for these categories + not using a continuous measure:

*Injection frequencies and frequency of equipment sharing were categorized into ordinal variables, rather than using continuous variables, to mitigate the impact of extreme outliers, address the highly skewed variable distribution, and to distinguish between key outcomes such as no injection days and daily injections (30 injections days).* (Lines 194-197)

*The ranges for the ordinal categories were chosen to proportionately capture the distribution of answers from all participants while highlighting no injection days and 30 injection days. The categories for injection days in the last 30 days are as follows: 1) 0 days (no injection), 2) 1–8 days (2 or less times a week), 3) 9–29 days, and 4) 30 days (daily injection). The categories for injection events in the last 30 days were categorized in the following: 1) 0 times (no injection), 2) 1–29 times (on average, less than once a day), 3) 30–89 times, and 4) 90+ times (on average, more than 3 times daily). The same ordinal categories were used for equipment sharing events in the last 30 days.* (Lines 201-208)

6. **Additional information regarding the retention and attrition by condition is needed along with how missing data was handled in the analysis.**

- Missing data, secondary to non-attendance at follow-up meetings, is a known limitation of this manuscript and this is recognized in the Limitations section. We ensured that the amount of lost data secondary to non-attendance was not significant between the two arms. All available information was used.
- The following has been added to the manuscript:

*The missingness of the data was also assessed via logistic regression for the five outcome variables at 12 months, employing the outcomes at the previous four data points as predictors.(24) This analysis aimed to examine if the patterns of missingness could be explained by the observed values. In addition, regardless of whether the missingness depended on the observed values, we conducted a sensitivity analysis using multiple imputation.* (Lines 221-225)

7. **Further description of modeling overall assessment points relative to examination at the 12-month follow-up is needed.**

Please see response to comment #6 above

8. **Table 2: Not clear what first 2 rows represent. This should be revised.**

Table 2 has been revised.

9. **The presentation of results in Table 3 should be revised.**

Table 3 has been edited to be more succinct, have less white space, and present the data more clearly.

10. **Figures 3 and 4 should indicate what the bars represent. Reviewing the figures it would appear while there may be a statistically significant difference, there may not be clinically meaningful differences between the two conditions. Further, these figures are somewhat misleading if the analysis utilized the ordinal categories rather than the continuous measures.**

The bars represent 95% confidence intervals, and a footnote has been added to the figures to clarify this.

The figures are meant to highlight the change in the number of people in clinically-relevant categories­­- especially in the “0 injection” category

11. **The authors list outliers as a limitation of this study. The rationale for ordinal categories is not well supported and it is unclear why the authors did not consider alternative analytic approaches.**

We have added the following sentence in the Methods section as a rationale for not using continuous measures and the thought process behind the ordinal categories used:

*Injection frequencies and frequency of equipment sharing were categorized into ordinal variables, rather than using continuous variables, to mitigate the impact of extreme outliers, address the highly skewed variable distribution, and to distinguish between key outcomes such as no injection days and daily injections (30 injections days).* (Lines 194-197)

*The ranges for the ordinal categories were chosen to proportionately capture the distribution of answers from all participants while highlighting no injection days and 30 injection days. The categories for injection days in the last 30 days are as follows: 1) 0 days (no injection), 2) 1–8 days (2 or less times a week), 3) 9–29 days, and 4) 30 days (daily injection). The categories for injection events in the last 30 days were categorized in the following: 1) 0 times (no injection), 2) 1–29 times (on average, less than once a day), 3) 30–89 times, and 4) 90+ times (on average, more than 3 times daily). The same ordinal categories were used for equipment sharing events in the last 30 days.* (Lines 201-208)

12. **There were a few instances where tracked changes remained in the document that should be addressed. Figure 1 did not appear correctly in the final submitted document.**

Figure 1 has been edited. Tracked changes have been removed.

**Additional Editor Comments**

**This is a randomized control trial of HCV syringe services conducted in New York.**

**The purpose of this study is quite interesting.**

1. **However, the methods are a bit confusing and the results are not very surprising or compelling.**

**The methods section has been revised in the following ways:**

- The differences in the AC and UC arms have been described in more detail to emphasize the differences between the two interventions
- The primary outcomes of the current study have been more explicitly stated
- Methods for statistical analysis have been clarified as per reviewers’ comments above

Our group believes that the results of the study are compelling and of clinical importance. Despite the pharmaceutical advancements allowing for a short and simple treatment course for HCV, PWID remain underdiagnosed and undertreated. Our study shows data highlighting the impact of an accessible, low threshold care model on not just SVR, but on injection behaviors that put PWID at risk for HCV in the first place. This is promising and important data for harm reduction for re-infections or infections with other blood-borne pathogens such as HIV. Our conclusion section goes into further detail regarding the importance of our findings both from a clinical and policy perspective.

2. **The terms “accessible” care versus “usual” care are not described in the abstract and require additional explanation for the casual reader.**

**Sentence introducing AC and UC in the methods section of the abstract has been revised to the following. Due to space limitation, the methods section was revised to describe the differences between the AC and UC arms in more detail:**

*Participants who were hepatitis C virus RNA positive and had injected drugs in the last 90 days were enrolled and randomized 1:1 to the an on-site, low threshold accessible care arm or a standard, referral-based usual care arm.* (Line 34-36)

3. **Similarly, the explanation in the methods requires additional details as given in Supplementary Figure 1.**

The methods section outlines the recruitment process, inclusion/exclusion criteria, and randomization process that is outlined in supplementary figure 1. The Results section has been edited to include the following:

*Between July 2017 and March 2020, 572 individuals were screened for eligibility. The most common reasons for exclusion were negative/no HCV RNA testing and no injection drug use within the last 90 days (Supplementary Figure 1). A total of 165 participants were enrolled with 82 participants assigned to the AC arm and 83 to the UC arm. (*Line 232-235)

4. **The authors should explain lines 145-146 in more detail:  The on-site treatment team facilitated a non-stigmatizing atmosphere during their interactions with the AC arm participants.**

The previous sentence has been revised to the following:

*The on-site team facilitated a non-stigmatizing atmosphere by maintaining a friendly and informal relationship with the participants and not pressuring for the adoption or rejection of any specific behaviors.* (Line 146-148)

5. **Lines 149-151:  is this information standardized in written / oral / virtual form?**

**Re-infection prevention sessions were interactive oral presentations that the care coordinators offered the AC participants receiving DAA therapy. The methods section has been edited to include this information:**

*Exclusive to the AC arm, all participants receiving DAA therapy were offered an on-site,*

*interactive re-infection prevention oral presentation adapted from the validated Staying Safe Intervention program.* (Line 153-155)

6. **It is unclear what drugs were injected and if that had any impact on injection behaviors, SVR, and/or study visits.**

- **Table 1 has been edited to include the types of drugs that were regularly used in the last 90 days**
- **We did not analyze whether the types of drugs regularly used at baseline had an impact on injection frequencies/behaviors. This was felt to be outside of the scope of this study as our primary focus was on AC vs. UC as the independent variables and their impact on injection frequency/behavior**
- **Our parent study showed that there was no impact on SVR rates dependent on the types of drugs regularly used at baseline**

7. **The data presented in Supplementary Figure 1 should be explained in more detail in the first paragraph of the Results.  More context about the cohort is needed.**

The following has been added to the result section to summarize the data in Supplementary Figure 1:

*Between July 2017 and March 2020, 572 individuals were screened for eligibility. The most common reasons for exclusion were negative/no HCV RNA testing and no injection drug use within the last 90 days (Supplementary Figure 1). A total of 165 participants were enrolled with 82 participants assigned to the AC arm and 83 to the UC arm. (*Line 232-235)
